# Cannabis Inflorescence Yield and Cannabinoid Concentration Are Not Increased With Exposure to Short-Wavelength Ultraviolet-B Radiation

**DOI:** 10.3389/fpls.2021.725078

**Published:** 2021-11-02

**Authors:** Victoria Rodriguez-Morrison, David Llewellyn, Youbin Zheng

**Affiliations:** School of Environmental Sciences, University of Guelph, Guelph, ON, Canada

**Keywords:** *Cannabis sativa*, potency, ultraviolet, indoor, sole source, terpene

## Abstract

Before ultraviolet (UV) radiation can be used as a horticultural management tool in commercial *Cannabis sativa* (cannabis) production, the effects of UV on cannabis should be vetted scientifically. In this study we investigated the effects of UV exposure level on photosynthesis, growth, inflorescence yield, and secondary metabolite composition of two indoor-grown cannabis cultivars: ‘Low Tide’ (LT) and ‘Breaking Wave’ (BW). After growing vegetatively for 2 weeks under a canopy-level photosynthetic photon flux density (PPFD) of ≈225 μmol⋅m^–2^⋅s^–1^ in an 18-h light/6-h dark photoperiod, plants were grown for 9 weeks in a 12-h light/12-h dark “flowering” photoperiod under a canopy-level PPFD of ≈400 μmol⋅m^–2^⋅s^–1^. Supplemental UV radiation was provided daily for 3.5 h at UV photon flux densities ranging from 0.01 to 0.8 μmol⋅m^–2^⋅s^–1^ provided by light-emitting diodes (LEDs) with a peak wavelength of 287 nm (i.e., biologically-effective UV doses of 0.16 to 13 kJ⋅m^–2^⋅d^–1^). The severity of UV-induced morphology (e.g., whole-plant size and leaf size reductions, leaf malformations, and stigma browning) and physiology (e.g., reduced leaf photosynthetic rate and reduced F_v_/F_m_) symptoms intensified as UV exposure level increased. While the proportion of the total dry inflorescence yield that was derived from apical tissues decreased in both cultivars with increasing UV exposure level, total dry inflorescence yield only decreased in LT. The total equivalent Δ^9^-tetrahydrocannabinol (Δ^9^-THC) and cannabidiol (CBD) concentrations also decreased in LT inflorescences with increasing UV exposure level. While the total terpene content in inflorescences decreased with increasing UV exposure level in both cultivars, the relative concentrations of individual terpenes varied by cultivar. The present study suggests that using UV radiation as a production tool did not lead to any commercially relevant benefits to cannabis yield or inflorescence secondary metabolite composition.

## Introduction

Drug-type *Cannabis sativa* (i.e., genotypes grown for their high cannabinoid content; hereafter, cannabis) are short-day plants commonly cultivated for their unique secondary metabolites (e.g., cannabinoids) that are used both medicinally and recreationally (Small, 2017). Cannabis is often grown in controlled-environment facilities that are illuminated solely with electrical lighting to accommodate its photoperiod specificity and produce uniform plants by maintaining prescribed environmental parameters (Zheng, 2021). Popular sole-source lighting technologies used in the flowering stage of cannabis production include high-pressure sodium (HPS) and, increasingly, light-emitting diodes (LEDs) (Cannabis Business Times, 2020).

Both HPS and LED technologies normally have little or no ultraviolet (UV; 100 to 400 nm) radiation in their spectra (Radetsky, 2018). Conversely, cannabis plants in the natural environment are exposed to a small but significant fraction of UV radiation relative to the amount of photosynthetically active radiation (PAR; 400-700 nm) in sunlight (Nikiforos et al., 2011). While the relative spectral distribution of UV and PAR wavebands varies over time and space, the UV waveband normally comprises approximately 5% of the day-time PAR intensity at any given time and global location. The solar UV that reaches the earth’s surface is comprised mostly of ultraviolet A (UVA; 315 to 400 nm) with the remainder being ultraviolet B (UVB; 280 to 315 nm) at an irradiance ratio of approximately 40:1 (Nikiforos et al., 2011), although reported UVA to UVB ratios range from 20:1 to 100:1, depending on time and place. The wavelength cutoff for solar UV reaching the earth’s surface is approximately 290 nm (Nikiforos et al., 2011), meaning that outdoor-grown plants are not exposed to short-wavelength UVB (i.e., <290 nm) or UVC (100 to 280 nm) (McElroy and Fogal, 2008). While UVC is used in horticultural applications to inactivate microorganisms such as waterborne pathogens in recirculating irrigation systems (Younis et al., 2019), foliage is only rarely directly exposed UVC – normally to inactivate foliar pathogens through short-term exposures (Aarrouf and Urban, 2020) – since UVC can cause tissue damage (Stapleton, 1992).

Many studies have investigated the effects of stratospheric ozone depletion on plant exposure to UV radiation (Searles et al., 2001; Caldwell et al., 2003) either through ecological or controlled-environment type research. The ratios between UV and PAR in controlled-environment type investigations tend to be much higher than in the solar spectrum in terrestrial ecosystems (Robson et al., 2019). Therefore plants in these studies may have exhibited relatively amplified responses to UV radiation including increased secondary metabolite accumulation and reduced photosynthesis and growth relative to lower UV:PAR responses (Behn et al., 2010; Dou et al., 2019). The other spectra within the lighting environment have also been shown to influence plant sensitivity to UV radiation, including biomass accumulation (Palma et al., 2021). Some spectra, such as UVA, have even been shown to counteract UVB-induced damage (Krizek, 2004). Perhaps through serendipity, researchers have discovered some potential horticultural benefits for providing unnaturally stressful UV exposure conditions which can enhance pertinent traits in economically relevant crops, for example increasing secondary metabolite concentrations (Huché-Thélier et al., 2016).

Relative to the UVA and PAR in the solar spectrum, the higher-energy photons in the UVB waveband are disproportionately effective in evoking plant responses, including changes in morphology, physiology, and metabolism (Flint and Caldwell, 2003; Huché-Thélier et al., 2016; Jenkins, 2017; Robson et al., 2019). Plant responses to UVB exposure are induced through pathways mediated by UV resistance 8 (UVR8; a UV-specific photoreceptor) or by UV-induced oxidative cellular damage, including to DNA (Czégény et al., 2016; Tossi et al., 2019). Typical plant responses to UV exposure include stunted growth, reduced leaf area, increased leaf thickness (Robson et al., 2019), epicuticular wax accumulation (Cen and Bornman, 1993), and foliar necrosis (Klem et al., 2012; Torre et al., 2012). From an ecological standpoint, it has been speculated that production of Δ^9^-tetrahydrocannabinol (Δ^9^-THC), which is the most economically valuable psychoactive cannabinoid, may be upregulated in cannabis tissues under UV exposure to serve as photoprotection. This concept arose from studies that found comparatively higher Δ^9^-THC concentrations in cannabis ecotypes that were grown in global regions with relatively high solar UV exposure, such as at low latitudes and high altitudes (Small and Beckstead, 1973; Pate, 1983). However, despite an apparent focus on interactions between UV and Δ^9^-THC in the cannabis literature, other cannabinoids have similar UV absorbing properties (Hazekamp et al., 2005), which may challenge an ecological explanation for favoring the upregulation of Δ^9^-THC over other cannabinoids in plants under UV stress.

Preliminary controlled-environment studies, that were done about three decades ago, also alluded to the potential for UV to increase Δ^9^-THC concentration in cannabis foliar and floral tissues (Fairbairn and Liebmann, 1974; Lydon et al., 1987). However, the concentration of Δ^9^-THC in mature female cannabis inflorescence tissues (hereafter, inflorescence) has increased substantially over the past decades, with contemporary genotypes having ≈10 times higher Δ^9^-THC concentrations than the genotypes used in these older studies (Dujourdy and Besacier, 2017). Therefore, modern cannabis genotypes may function nearer to cannabis’ maximum capacity for producing Δ^9^-THC; which could impede their ability to further increase Δ^9^-THC production under an abiotic stress such as UV exposure, relative to older genotypes. However, studies on modern cannabis genotypes have shown that various environmental stimuli can modify the cannabinoid composition. For example, inflorescences of cannabidiol (CBD)-dominant genotypes had greater CBD concentrations when grown at high vs. low altitude, which may have been a response to increased UV exposure at higher elevation (Giupponi et al., 2020). Drought-stress, salt-stress, and PAR spectra have also been shown to alter the inflorescence cannabinoid composition in modern, indoor-grown genotypes (Mahlberg et al., 1983; Magagnini et al., 2018; Caplan et al., 2019; Yep et al., 2020; Westmoreland et al., 2021). Therefore, the potential for UV exposure to provoke changes in the secondary metabolite composition in inflorescences of modern cannabis genotypes grown in controlled environments merits scientific investigation. Evaluating the effects of UV on modern genotypes with relatively balanced concentrations of Δ^9^-THC and CBD [i.e., chemotype II; a cultivar with a ratio of Δ^9^-THC to CBD of ≈1 (Small and Beckstead, 1973)] may provide additional insight into cannabinoid-specificity of UV exposure responses.

The objectives of this study were to: (1) characterize morphological and physiological responses of indoor-grown cannabis to UV exposure, and (2) investigate the relationships between UV exposure levels applied during the flowering stage and inflorescence yield and secondary metabolite composition of modern chemotype II cannabis genotypes.

## Materials and Methods

### Plant Culture

Clonal cuttings were taken from mother plants of indoor grown cultivars: ‘Low Tide’ and ‘Breaking Wave’ and allowed to root for 13 d under humidity domes and fluorescent light (F32T8/TL850; Philips, Amsterdam, Netherlands) providing a photosynthetic photon flux density (PPFD, 400 to 700 nm) of ≈100 μmol⋅m^–2^⋅s^–1^ at the canopy. Rooted cuttings were transferred to 3.79L pots (height: 18.4 cm, diameter: 16.2 cm) containing a peat-based substrate and grown for an additional 9 d under LED light comprised of a mixture of Pro-325 (Lumigrow; Emeryville, CA, United States) and generic (unbranded) white LEDs providing a PPFD of ≈225 μmol⋅m^–2^⋅s^–1^ at the canopy. The propagation and vegetative growth stages both had 18-h photoperiods. The potted plants were subsequently transferred to a single deep-water culture basin (CB), where they were placed in floating polystyrene rafts in an indoor cannabis production facility in southern Ontario, Canada (described in Rodriguez-Morrison et al., 2021a). There were 384 evenly-spaced plants in the CB at a density of 0.09 m^2^/plant. The daily PAR photoperiod was reduced to 12 h (07:30 HR to 19:30 HR) on the day the plants were transferred to the CB.

### PAR and UV LED Fixture Layout

**FIGURE 1:**
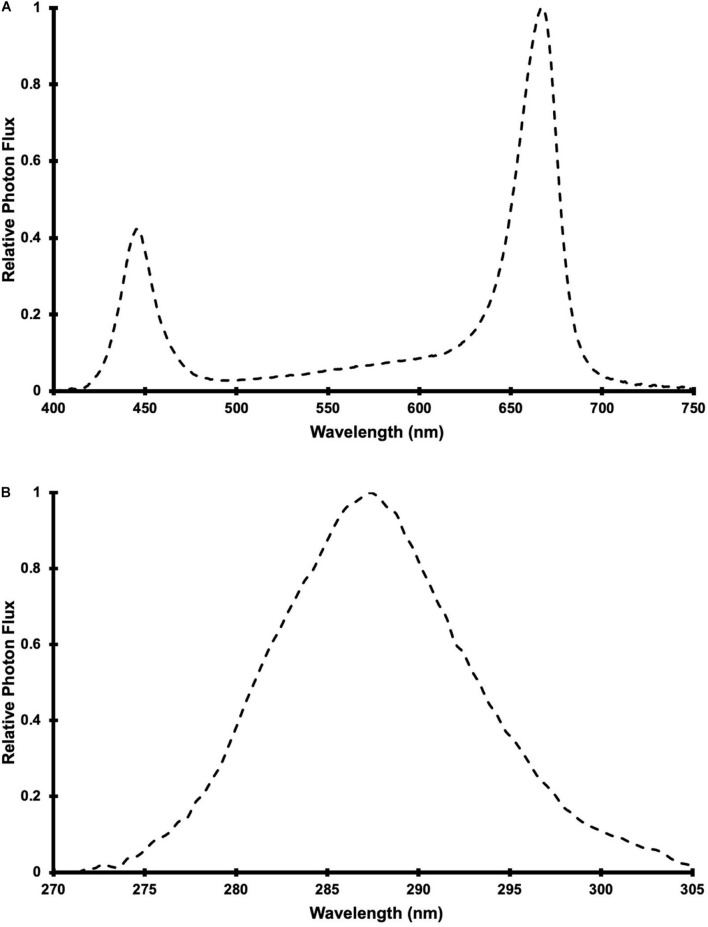
Relative spectral photon flux distribution of **(A)** Pro-650 Lumigrow LED fixtures and **(B)** UV LED fixtures.

Photosynthetically active radiation was supplied by 24 LED fixtures (Pro650; Lumigrow Inc.) arranged evenly over the CB in 2 rows of 12 fixtures. The LED composition and spectrum of the PAR fixtures were described in Rodriguez-Morrison et al. (2021a) and the relative spectral photon flux distribution is provided in Figure 1A. Single UV LED fixtures were centered between adjacent PAR fixtures (within each row), resulting in 2 rows of 11 UV fixtures. The 22 UV LED fixtures were a custom design (10 × 90 cm), comprised of UVB LEDs with a peak wavelength of 287 nm (Figure 1B) and adjustable intensity (with analog, constant current dimmers). According to the conventional definitions of the different UV wavebands, the photon flux ratio of UVB (280 to 315 nm) to UVC (100 to 280 nm) was UVB(93):UVC(7). Additionally, 30% of the UV photon flux was at wavelengths >290 nm and there was no photon flux between 310 and 400 nm or <270 nm. The UV treatments (described below) were applied daily, in the last 3.5 hours (16:00 HR to 19:30 HR) of the PAR photoperiod, for 60 d from the day that the plants were transferred to the CB and then harvested.

### Experimental Setup

The experiment was arranged and carried out as a gradient design, which can outperform treatment × replication designs when evaluating biological responses along a continuous independent variable (Kreyling et al., 2018), such as radiation intensity (Rodriguez-Morrison et al., 2021a). With a gradient design, regression analyses are performed on the response variables (i.e., measured parameters) against all different levels of the independent variable.

**FIGURE 2:**
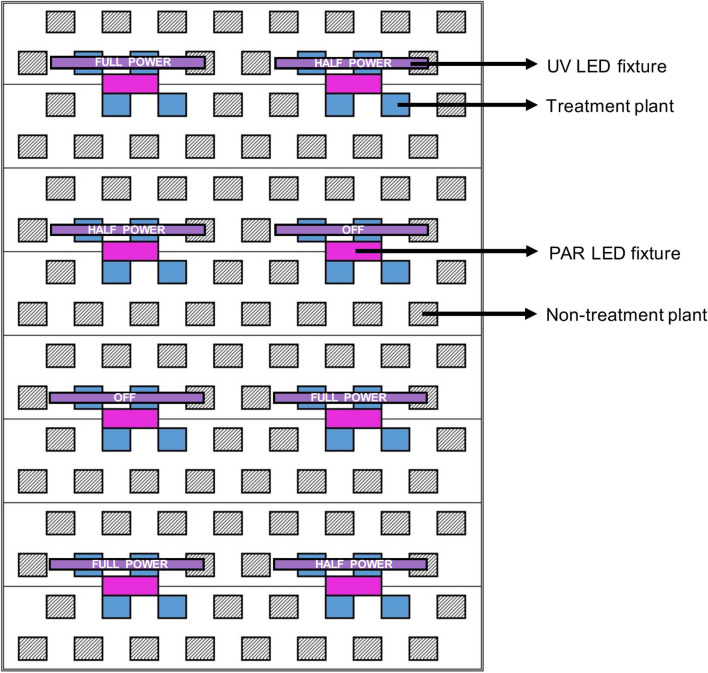
Schematic of a single LED rack comprised of 8 PAR fixtures (in magenta) and 8 UV fixtures (in purple) above one third of the deep-water culture basin (CB). The entire growing area consisted of 3 light racks. The 3 UV LED settings (off, half power, full power) were randomly assigned to individual UV fixtures (i.e., plots). Each treatment plant (in blue, 4 per plot) was assigned a UV exposure level, reflecting its average canopy-level UV photon flux density (UV-PFD) measured throughout the trial. The UV-PFDs were used as the independent variable in analyses of plant growth, physiology and harvest metrics. Each plot was surrounded by non-treatment plants (hatched diagonal lines) to ensure uniform growing environment and normal planting density. The north half of the CB was populated with BW and the south half was populated with LT.

For each cultivar, 44 uniform representative plants were selected from the larger populations to be experimentally evaluated. Plots, each consisting of 4 plants of a single cultivar, were arranged where 2 plants were positioned directly below each UV LED fixture, and 2 plants were adjacent. Three UV LED fixture settings were randomly assigned (within each cultivar) to each plot: off, half power, and full power. Within each plot, the 2 plants positioned below the UV LED fixtures had relatively higher UV exposure than the 2 adjacent treatment plants. This configuration allowed for each cultivar to be exposed to a wide array of UV photon flux densities (UV-PFD); evenly-distributed across the 0.01 to 0.8 μmol⋅m^–2^⋅s^–1^ range (Figure 2).

At the start of the UV treatments, experimental plants of LT had heights from the substrate surface to the shoot apex ranging from 14 to 23 cm and experimental plants of BW had heights ranging from 14 to 20 cm. Experimental plants were surrounded by plants of the same cultivar to maintain canopy uniformity. The LT cultivar populated the south half of the CB, while BW populated the north half.

The average distance from the bottom of the fixtures to the top of the treatment plants was maintained at 50.5 cm by adjusting the height of the light racks weekly using a system of pulleys and cables. Canopy-level PPFD and UV-PFD were measured at the apex of each plant weekly, after the light rack height adjustment, using a PAR meter (LI-180; LI-COR Biosciences, Lincoln, NE, United States) and a radiometrically calibrated spectrometer (UV-Vis Flame-S-XR, Ocean Optics, Dunedin, Florida), respectively. The UV-PFDs were measured with the PAR LEDs turned off. A MS Excel tool developed by Mah et al. (2019) was used to integrate spectrometer data into UV-PFD, biologically-effective UV-PFD (Flint and Caldwell, 2003), and daily biologically-effective UV dose (kJ⋅m^–2^⋅d^–1^) (Table 1). At the end of the trial, average PPFD and average UV-PFD were calculated for each treatment plant by determining the corresponding total light integrals (TLI; mol⋅m^–2^) and then dividing by accumulated time, as described in Rodriguez-Morrison et al. (2021a). The experiment-wise average (± SE, *n* = 88) PPFD was 408 ± 6.5 μmol⋅m^–2^⋅s^–1^. The average UV-PFD for each plant was used as the independent variable (i.e., x-axis) in regressions of UV exposure vs. the measured growth, yield and quality parameters.

**TABLE 1:** Range of canopy-level UV photon flux density, UV biologically-effective photon flux density and daily UV biologically-effective dose from UV LEDs with a peak wavelength of 287 nm and a daily 3.5 h photoperiod.

UV exposure level	UV photon flux density (μmol⋅m^–2^⋅s^–1^)	UV biologically-effective^*z*^ photon flux density (μmol⋅m^–2^⋅s^–1^)	Daily UV biologically- effective dose (kJ⋅m^–2^⋅d^–1^)
Minimum	0.01	0.032	0.16
Low	0.1	0.32	1.6
Moderate	0.5	1.6	8.0
Maximum	0.8	2.6	13

*^z^Weighted using the Biological Spectral Weighting Factor for Plant Growth by Flint and Caldwell (2003).*

Plant husbandry and environmental controls followed the cultivator’s standard operating procedures except for the UV radiation. The air temperature and relative humidity set points were 25°C and 60%. There was no CO_2_ supplementation, with typical concentrations of ≥400 ppm when the PAR lights were on. Air was continuously circulated throughout the room with wall-mounted axial fans and the HVAC circulation rate was ≈2 air changes per hour (ACH) with ≥25% refresh with pre- conditioned outside air. The aquaponic solution was maintained within normal levels of nutrient concentrations, pH, electrical conductivity, and dissolved oxygen; as described in Rodriguez-Morrison et al. (2021a).

### Growth Measurements

The height (i.e., length of main stem from substrate surface to the highest point) and widths (i.e., the widest part and its perpendicular width) of each experimental plant were measured in week 6. Plant height and widths were used to calculate growth index [(height × width_1_ × width_2_)/300 (Ruter, 1992)] for each plant, where width_1_ was the widest part of the plant and width_2_ was perpendicular to width_1_.

### Leaf Chlorophyll and Fluorescence Measurements

Foliar chlorophyll content index [CCI; the ratio of % transmission 569 at 931 nm and % transmission at 653 nm (Parry et al., 2014)] was measured in upper and lower canopy leaves in week 3. Measurements of CCI were taken from the center leaflet of the three youngest fully-expanded fan leaves and from three fan leaves at the bottom of each plant using a chlorophyll meter (CCM-200; Opti-Sciences, Hudson, NH, United States). The CCI measurements were averaged, for the upper and lower canopy leaves, respectively, on a per plant basis.

The ratio of variable to maximum fluorescence (F_v_/F_m_) emitted from photosystem II in dark-acclimated leaves exposed to a light-saturating pulse is an indicator of maximum quantum yield of photosystem II photochemistry (Murchie and Lawson, 2013). In week 5, during the first 8.5 h of the PAR photoperiod (i.e., before daily UV exposure), the middle leaflet of the youngest fully-expanded fan leaf from each plant was dark acclimated for 15 min and then F_v_/F_m_ measurements were taken with a fluorometer (FluorPen FP 100; Drasov, Czech Republic).

### Leaf Gas Exchange, Leaf Size and Specific Leaf Weight

Quantifications of leaf gas exchange of the middle leaflet of the youngest, fully-expanded fan leaf on each treatment plant was performed in week 5 during the first 8.5 h of the PAR photoperiod using a portable photosynthesis machine (LI-6400XT; LI-COR Biosciences, Lincoln, NE, United States) equipped with the B and R LED light source (6400-02B; LI-COR Biosciences). *In situ* net CO_2_ exchange rate (NCER) was measured with the leaf cuvette environmental conditions set to: PPFD of 500 μmol⋅m^–2^⋅s^–1^, block temperature of 26.7°C, CO_2_ concentration of 400 ppm, and air flow rate of 500 μmol⋅s^–1^. Because the leaflets were not wide enough to cover the entire cuvette, the section of each leaflet that was clamped in the cuvette gasket was marked along the outside of the gasket with a permanent marker so that leaf area inside the cuvette could be calculated *post hoc* (described below). After removing the leaflets from the cuvette, whole leaves were excised from the plant and scanned (CanoScan LiDE 25; Canon Canada Inc., Brampton, ON, Canada) at 600 dpi resolution. Each leaf was oven dried to constant weight at 65°C (Isotemp Oven 655G; Fisher Scientific, East Lyme, CT, United States). The scanned images were processed using ImageJ 1.42 software (National Institute of Health; https://imagej.nih.gov/ij/download.html) to determine the leaflet area within the gas exchange chamber (by subtracting the width of the chamber gaskets from the marks made during gas exchange measurements) and the total individual leaf size. The NCER for each leaf was corrected for measured leaf area inside the cuvette. The dry weight (DW) of each entire scanned leaf was measured using an analytical balance (MS304TS/A00; Mettler-Toledo, Columbus, OH, United States) to determine specific leaf weight [SLW; leaf DW/leaf size (g⋅m^–2^)].

### Visual Observations

Weekly observations were performed on each entire plant to visually evaluate observable changes in morphological attributes, including: upward curling of the leaflet margins, leaf shine, browning of stigmas, leaf epinasty, leaf necrotic patches, and appearance of powdery mildew on the adaxial sides of the leaves. Except for week 1 observations, which occurred 4 d after the start of the UV treatments, all weekly observations occurred on 7-d intervals thereafter. The absence or presence of each respective parameter was evaluated for each plant weekly, except where noted in the results (Table 2). While these are observational data, the minimum UV-PFDs under which individual attributes were observed were reported, on per cultivar and per week bases, regardless of whether all plants exposed to higher UV-PFDs displayed the observed responses.

**TABLE 2:** Minimum UV-PFD (μmol⋅m^–2^⋅s^–1^) where symptoms were observed in *Cannabis sativa* ‘Low Tide’ (LT) and ‘Breaking Wave’ (BW) cultivars in each week after the initiation of UV treatments, regardless of whether all plants above the minimum UV-PFD presented the observed symptom.

		Foliar			Inflorescence
			
Week^z^	Cultivar	Upward curling	Epinasty	Necrotic patches	Stigma browning
1	LT	0.33	NI^y^	NI	NI
1	BW	0.37	NI	NI	NI
2	LT	NIE^x^	NI	NI	NI
2	BW	NIE	NI	NI	NI
3	LT	NIE	NI	NI	0.69
3	BW	NIE	NI	NI	0.30
4	LT	NIE	NI	NI	0.22
4	BW	NIE	NI	NI	0.14
5	LT	0.16	0.13	NI	0^w^
5	BW	0.34	0.14	NI	NIE
6	LT	0.13	NIE	NI	0
6	BW	0.33	NIE	NI	0
7	LT	NIE	0.10	0.12 to 0.69^v^	0
7	BW	NIE	0.13	0.32	0
8	LT	NIE	0.018	0.12 to 0.70	0
8	BW	NIE	0.034	0.20 to 0.51	0
9	LT	NIE	NIE	NIE	0
9	BW	NIE	NIE	NIE	0

*^z^All weekly observations occurred on 7-d intervals except for observations in week 1, which occurred 4 days after the start of the UV treatments.*

*^y^NI: symptom was not investigated.*

*^x^NIE: no increase in extent of crop sensitivity to UV exposure level was observed.*

*^w^Zero indicates that the symptom was observed at the lowest UV-PFD.*

*^v^Ranges are provided when the symptom was observed in only intermediate UV-PFDs.*

At various points throughout the trial, photos of representative whole plants and specific tissues of each cultivar growing under different UV exposure levels were taken with a digital camera (iPhone XR iOS 14.4.1; Cupertino, CA, United States) or flat bed scanner (CanoScan LiDE 25). Photos of whole plants grown under minimum and maximum UV exposure levels were taken in week 2. Photos of the inflorescences grown under minimum and maximum exposure levels, of each cultivar, were taken in week 3. Photos of whole plants grown under minimum, low, moderate and maximum UV exposure levels (described in Table 1) were taken in week 3. In week 5 [i.e., approximately when vegetative growth in cannabis ceases (Rodriguez-Morrison et al., 2021a)], fully-expanded leaves from plants under minimum, moderate, and high UV exposure were excised from the plants and scanned at 600 dpi resolution. Photos of whole plants and apical inflorescences grown under minimum, low, moderate, and maximum UV exposure levels were taken at harvest (i.e., week 9). All photos were processed using ImageJ 1.42 software to add appropriate scale bars.

### Yield and Quality

After 60 d of UV exposure, all treatment plants were harvested by cutting the stems at substrate level. The LEDs were turned off prior to harvest and plants were harvested, randomly, one at a time to minimize any harvesting effects on fresh biomass assessments. The aboveground tissue of each plant was separated into stems, leaves, and inflorescences. The inflorescences were further subdivided into apical (i.e., grouping of terminal inflorescences at the top of the main stem, located above the uppermost side-branch) and non-apical groupings. All inflorescence tissues were trimmed of foliar materials, according to the cultivator’s normal practices. The fresh weights (FW) of stems, leaves, and apical and non-apical inflorescence were separately recorded for each plant using a precision balance (EG 2200-2NM; Kern, Balingen, Germany). The apical inflorescences for 18 plants of each cultivar that were representative of the entire range of UV-PFD exposure levels were air dried at 15°C and 40% relative humidity for 7 d. Following air drying, ≈2 g sub-samples (actual weights were recorded) from each plant were submitted to an independent laboratory (RPC Science & Engineering; Fredericton, NB, Canada) for analysis of concentrations [reported in mg⋅g^–1^_(DW)_] of cannabinoids using ultra-high-performance liquid chromatography and variable wavelength detection (HPLC-VWD), terpenes using gas chromatography and mass spectrometry detection (GC-MSD), and moisture content. The total equivalent (annotated with: *eq*) Δ^9^-THC, CBD, and cannabigerol (CBG) concentrations were determined by assuming complete carboxylation of the acid-forms of the respective cannabinoids, whose concentrations were adjusted by factoring out the acid-moiety from the molecular weight of each respective compound [e.g., Δ^9^-THC*eq* = (Δ^9^-THCA × 0.877) + Δ^9^-THC]. The remaining apical tissues from the air-dried samples and the entire apical inflorescences of the non-air-dried plants were re-combined with non-apical inflorescences to make up total inflorescence groupings, on a per-plant basis. All separated aboveground tissues of all plants were oven-dried at 65°C to constant weight (Isotemp Oven 655G) and the DW of the respective tissues were recorded. Moisture content of each separated aboveground tissue grouping was calculated as: [(FW – DW) / FW] × 100%. The sub-sampled apical tissues were accounted for in this calculation using their respective sample weight and moisture content measurements for each sample, provided by RPC.

### Statistical Analysis

The UV-PFD in this experiment was a continuous, independent variable based on the weekly calculated UV-PFDs for each individual plant. On a per cultivar bases, the best-fit models (linear, quadratic, or cubic) for measured parameters vs. UV-PFD were selected based on the lowest value for the Akaike Information Criterion (AICc) using UV-PFD as the independent variable using the PROC NLMIXED procedure (SAS Studio Release 3.8; SAS Institute Inc., Cary, NC, United States). Analyses also revealed that each dataset had a normal distribution. For parameters that were measured prior to harvest, the average UV-PFD for each plant determined based on the weekly UV measurements made until the parameter was measured. If there were no UV-PFD treatment effects on a given parameter, then parameter means (± SD) were calculated.

## Results

The canopy-level average UV-PFDs ranged from 0.01 to 0.8 μmol⋅m^–2^⋅s^–1^ for both cultivars; therefore, this range was used to contextualize the models presented below (e.g., lowest vs. highest UV-PFD) for all measured parameters. The average (± SE, *n* = 44) increases in UV-PFD between adjacent UV-PFD levels was 2.3 ± 0.46% for LT and 2.3 ± 0.39% for BW. The photon flux ratio of PAR to UV at the maximum UV-PFD was ≈500:1, which was within the range normally reported for PAR to UVB in the solar spectrum.

### UV-Induced Cannabis Morphology and Physiology Changes

While the aboveground portions of each entire plant was observed for UV-induced changes in morphology, the recorded effects occurred primarily in recently-developed tissues. When UV effects were also seen in older tissues, this has been highlighted in the text. The data in Table 2 are provided to show how the development of temporal trends in these observed parameters related to each other, and how crop sensitivity to UV exposure increased over time.

**FIGURE 3:**
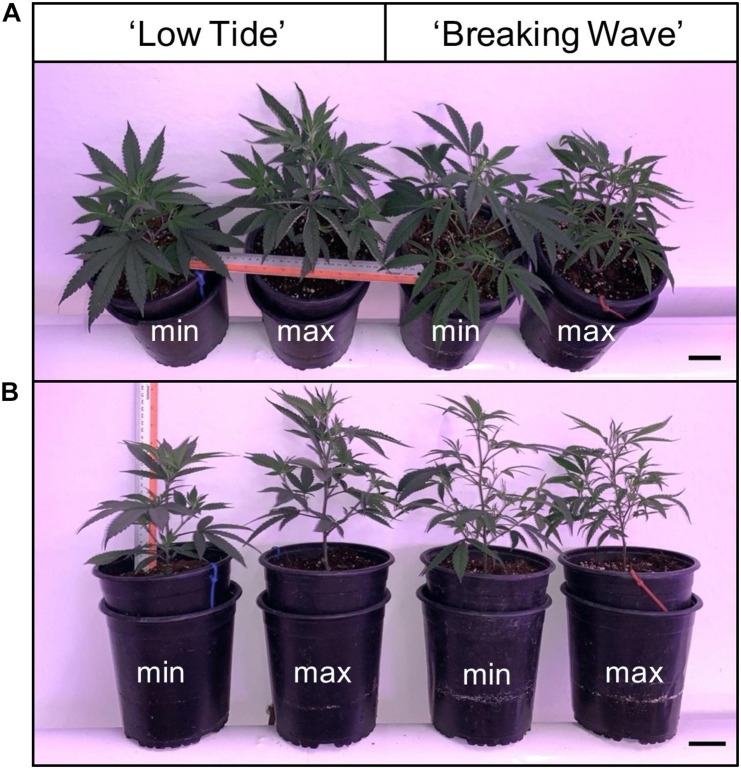
**(A)** Side and **(B)** top views of representative *Cannabis sativa* plants of ‘Low Tide’ and ‘Breaking Wave’ under minimum (min) and maximum (max) UV exposure levels in week 2 after the initiation of the UV treatments. The black scale bar at the lower right of each image is 5.0 cm.

**FIGURE 4:**
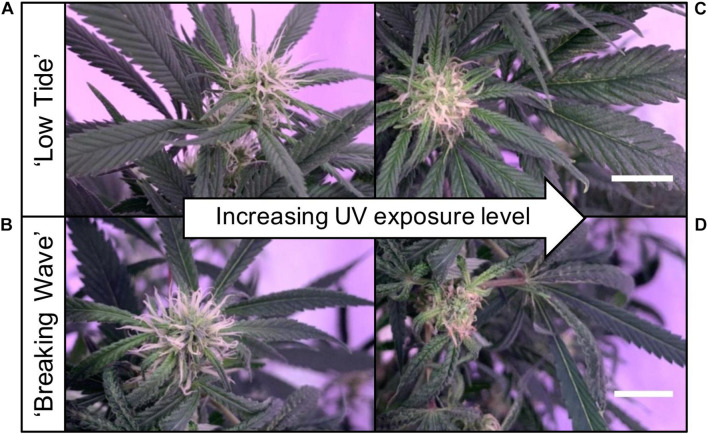
Images of stigmas of representative **(A)** ‘Low Tide’ (LT) and **(B)** ‘Breaking Wave’ (BW) *Cannabis sativa* plants grown under minimum UV exposure levels and **(C)** LT and **(D)** BW under maximum UV exposure levels, in week 3 after the initiation of the UV treatments. The white scale bar at the lower right of **(C)** applies to **(A)**, and at the lower right of **(D)** applies to **(B)**. Both scale bars are 1.0 cm.

**FIGURE 5:**
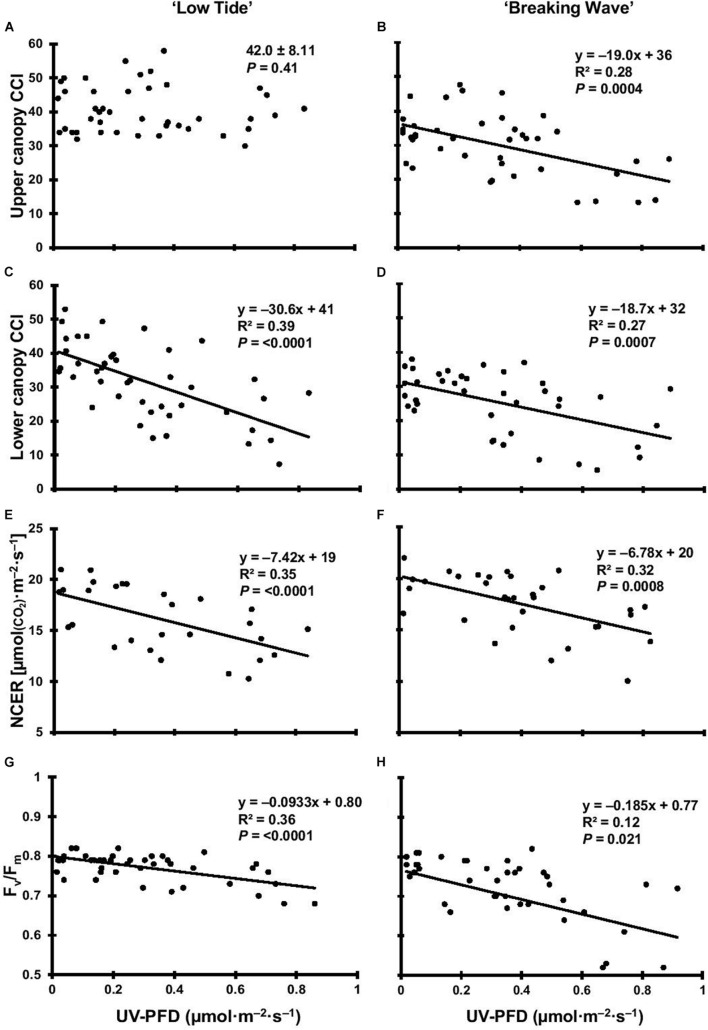
The upper canopy leaf chlorophyll content index (CCI) of *Cannabis sativa* ‘Low Tide’ **(A)** and ‘Breaking Wave’ **(B)**, the lower canopy leaf CCI of ‘Low Tide’ **(C)** and ‘Breaking Wave’ **(D)**, the net CO_2_ exchange rate (NCER) of ‘Low Tide’ **(E)** and ‘Breaking Wave’ **(F)**, and the F_v_/F_m_ of ‘Low Tide’ **(G)** and ‘Breaking Wave’ **(H)** in response to increasing UV-PFD. Each datum is a single plant.

**FIGURE 6:**
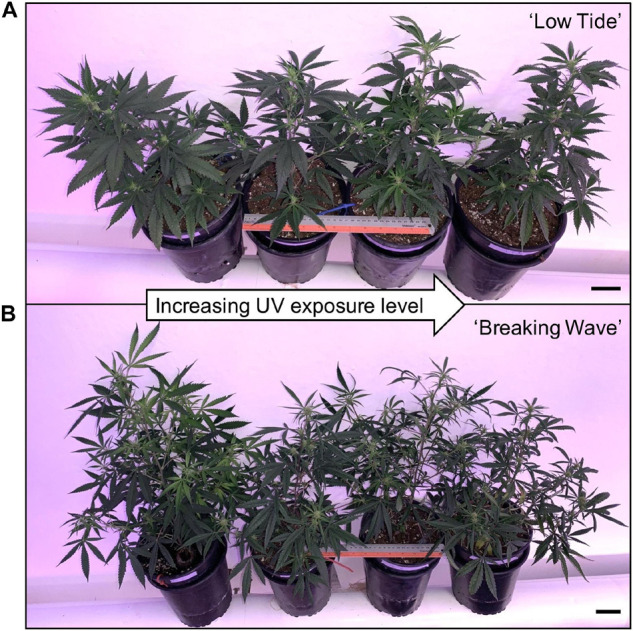
Representative **(A)** ‘Low Tide’ and **(B)** ‘Breaking Wave’ *Cannabis sativa* plants demonstrating (from left to right) minimum, low, moderate, and high UV exposure levels. The images were taken in week 3 after the initiation of the UV treatments. The black scale bar at the lower right of each image is 5.0 cm.

**FIGURE 7:**
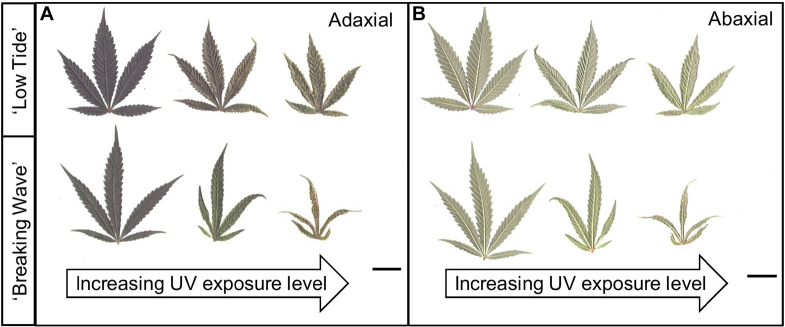
**(A)** Adaxial and **(B)** abaxial sides of representative youngest, fully-expanded *Cannabis sativa* fan leaves of ‘Low Tide’ (top row in each image) and ‘Breaking Wave’ (bottom row in each image) demonstrating UV induced leaf morphology effects with increasing UV-PFD. Leaves from plants under minimum UV exposure are on the left, moderate UV exposure in the middle, and high UV exposure on the right. Scans were taken in week 5 after the initiation of UV treatments. The black scale bar at the lower right of each image is 2.0 cm.

**FIGURE 8:**
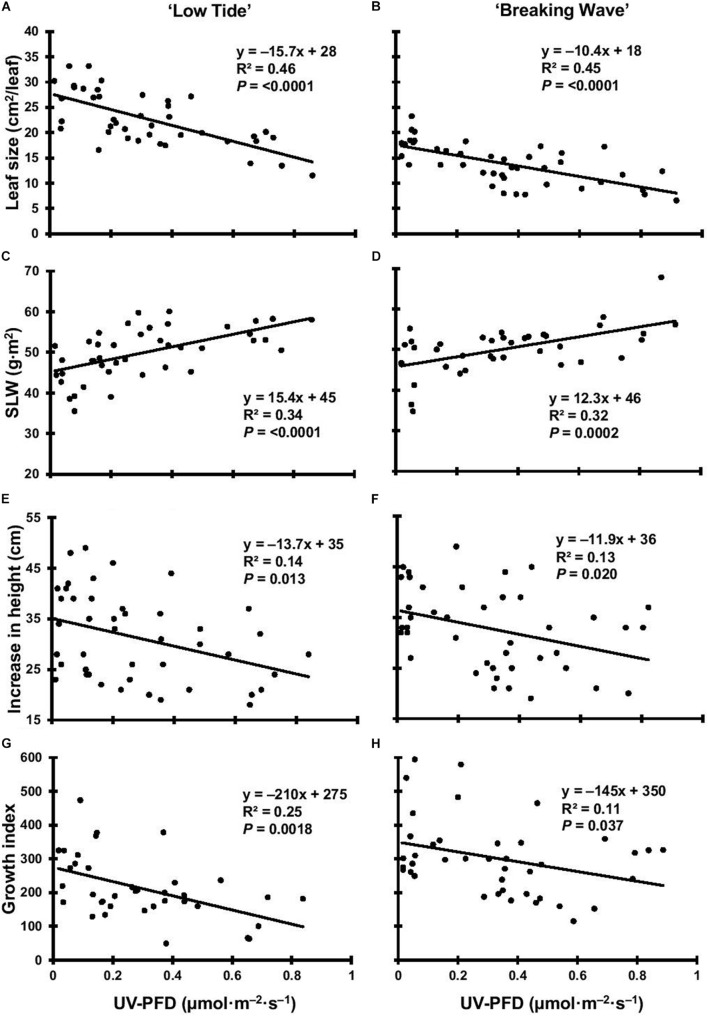
The leaf size of *Cannabis sativa ‘*Low Tide’ **(A)** and ‘Breaking Wave’ **(B)**, the specific leaf weight (SLW) of ‘Low Tide’ **(C)** and ‘Breaking Wave’ **(D)**, the increase in height of ‘Low Tide’ **(E)** and ‘Breaking Wave’ **(F)** and the growth index of ‘Low Tide’ **(G)** and ‘Breaking Wave’ **(H)** in response to increasing UV-PFD. Each datum is a single plant.

**FIGURE 9:**
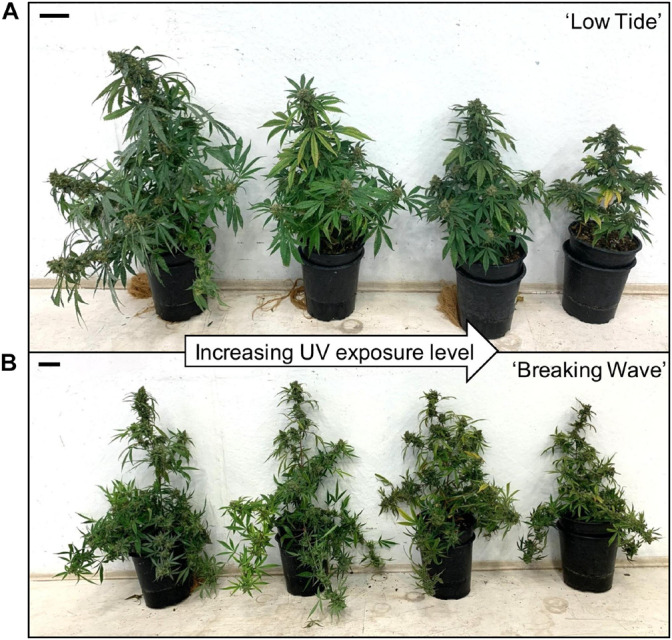
Gross plant morphology of representative **(A)** ‘Low Tide’ and **(B)** ‘Breaking Wave’ *Cannabis sativa* plants grown under (from left to right) minimum, low, moderate, and high UV exposure levels. Images were taken just prior to harvest (i.e., 9 weeks after the initiation of UV treatments). Note the white spots (powdery mildew) on the adaxial sides of leaves on the far-left plants in both images. The black scale bar at the upper left of each image is 5.0 cm.

The first observed UV-induced changes in cannabis morphology appeared within the first few days of the initiation of the UV treatments where the leaflet margins on leaves that had developed in the vegetative stage (i.e., prior to the initiation of the UV exposure) curled upwards under UV-PFDs ≥0.33 and ≥0.37 μmol⋅m^–2^⋅s^–1^ in LT and BW, respectively during week 1 (Table 2). Leaves also appeared to accumulate epicuticular wax, as demonstrated by the increase in shiny appearance of adaxial surfaces, shortly after UV exposure began and persisted henceforth (data not shown). Leaf shine appeared to be more prevalent in plants exposed to higher UV-PFDs, and the prevalence appeared to be greater in BW vs. LT. In week 2 there were no changes in the extent of upward curling in mid-canopy foliage (i.e., leaves that had developed during the vegetative stage) however, newly expanded leaves did not appear to present this symptom with the same level of severity (Figure 3). In week 3, which was about one week after the presence of inflorescence tissues were readily apparent, stigmas of terminal inflorescences began to turn from white to brown on LT and BW plants exposed to UV-PFDs ≥0.69 and ≥0.30 μmol⋅m^–2^⋅s^–1^, respectively (Figure 4 and Table 2). In week 3, early symptoms of foliar epinasty (i.e., interveinal tissues that were raised in the middle) started to appear in upper canopy leaves only of plants grown under higher UV-PFDs (Figure 4). Some leaves at the bottom of the plants started to become yellow and drop off in week 3 for both cultivars under higher UV-PFDs (data not shown). Fallen leaves appeared to be predominantly the same leaves that showed upward curling in week 1. There were no UV treatment effects on the CCI of the upper canopy leaves of LT in week 3, but the CCI of the upper canopy leaves of BW decreased linearly by 42% from the lowest to highest UV-PFD (Figures 5A,B). The CCI in the lower canopy leaves decreased linearly by 60% and 46% from the lowest to highest UV-PFD in LT and BW, respectively (Figures 5C,D). In week 4, the minimum UV-PFD at which plants exhibited stigma browning were lower than the previous week (Table 2). In week 5, the *in situ* NCER of the youngest fully-expanded leaves decreased linearly with increasing UV exposure, with 31% and 27% lower NCER at highest vs. lowest UV-PFD in LT and BW, respectively (Figures 5E,F). In week 5, the F_v_/F_m_ values of the youngest fully-expanded leaves decreased linearly by 9% and 19% in LT and BW, respectively (Figures 5G,H). The severity of UV-induced epinasty was elevated in plants exposed to higher UV exposure levels, as shown in images of whole plants in week 3 (Figure 6) and single-leaf scans in week 5 (Figure 7) of representative plants grown under different UV exposure levels. In week 5, upper canopy leaves in particular showed upward curling under UV-PFDs ≥0.16 and ≥0.34 μmol⋅m^–2^⋅s^–1^ in LT and BW, respectively (Table 2 and Figure 7). In week 5, brown stigmas were observed on plants grown under the lowest UV-PFD in LT and ≥0.14 μmol⋅s⋅m^–2^ in BW (Table 2). In week 5, leaf epinasty was predominantly evident in youngest fully-expanded leaves in plants exposed to UV-PFDs ≥0.13 μmol⋅m^–2^⋅s^–1^ in LT and ≥0.14 μmol⋅m^–2^⋅s^–1^ in BW (Table 2). In week 5, the size of the youngest fully-expanded leaves decreased linearly with increasing UV-PFD, with ≈45% reductions in young leaf size in plants grown under highest vs. lowest UV-PFD (Figures 8A,B). There were corresponding linear increases in SLW with increasing UV exposure, with 27% and 21% increases in LT and BW, respectively, in plants grown under the highest vs. lowest UV-PFD (Figures 8C,D). Brown stigmas were observed in all experimental plants starting in week 6 (Table 2). Starting in week 7, upper canopy leaves on a few LT plants grown under intermediate UV-PFDs, ranging from 0.12 to 0.69 μmol⋅m^–2^⋅s^–1^, began to show brown (necrotic) patches (Table 2). The minimum UV-PFDs under which leaf epinasty was evident were marginally lower in week 7 vs. week 5, and substantially lower in week 8 vs. week 7 (Table 2) in both cultivars. The prevalence of leaves exhibiting necrotic patches increased in BW in week 8 vs. week 7 (Table 2). Investigating the effects of UV exposure on foliar powdery mildew was not one of the objectives at the outset of this study, however, some potential treatment effects were observed. For example, evidence of powdery mildew was visible on the adaxial leaf surfaces on plants exposed to low UV-PFDs at harvest, but was not observed on any plants exposed to UV-PFDs ≥0.090 μmol⋅m^–2^⋅s^–1^ (Figure 9). By harvest, there also appeared to be greater incidences of foliar chlorosis in both cultivars, especially in the lower canopy, with increasing levels of ultraviolet exposure (Figure 9).

### Growth Responses to UV

Exposure to UV generally suppressed plant growth, which was recorded as increase in height and growth index in week 6. The majority of vegetative growth had ceased by week 6 given that height only increased 1.76 ± 0.41 cm and 0.62 ± 0.48 cm (mean ± SE) for LT and BW respectively, between week 6 and harvest (data not shown). Both increase in height and growth index had negative linear relationships with increasing UV-PFD in both cultivars. Increases in height were 31% and 26% lower in plants grown under the highest vs. lowest UV-PFDs in LT and BW, respectively (Figures 8E,F). Growth indices were 61% and 33% lower in plants grown under the highest vs. lowest UV-PFDs in LT and BW, respectively (Figures 8G,H). There were no UV treatment effects on the moisture content of any aboveground tissues in either cultivar (Table 3).

**TABLE 3:** The effects of UV-PFD (μmol⋅m^–2^⋅s^–1^) applied during the flowering stage on aboveground tissue moisture content (%), stem dry weight (DW; g⋅m^–2^), and cannabinoid and terpene concentrations [mg⋅g^–1^_(DW)_] in the mature, air-dried apical inflorescence tissues of *Cannabis sativa* ‘Low Tide’ and ‘Breaking Wave.’

Parameter	‘Low Tide’		‘Breaking Wave’	
	Regression equation^z^, *R*^2^ or mean ± SD^y^	*P*-value	Regression equation, *R*^2^ or mean ± SD	*P*-value
Inflorescence moisture content	79.0 ± 1.27	0.14	79.3 ± 1.01	0.37
Leaf moisture content	69.5 ± 3.59	0.94	72.0 ± 2.31	0.28
Stem moisture content	72.4 ± 2.93	0.77	74.5 ± 2.17	0.41
Stem DW	49.2 ± 26.5	0.44	46.1 ± 21.6	0.16
Δ^9^-tetrahydrocannabinol (Δ^9^-THC)	1.66 ± 0.347	0.18	y = 1.02x + 1.4, 0.33	0.013
Δ^9^-THC acid (Δ^9^-THCA)	y = −15.9x + 84, 0.29	0.020	73.8 ± 12.2	0.91
Cannabidiol (CBD)	1.38 ± 0.447	0.56	1.47 ± 0.338	0.12
CBD acid (CBDA)	y = −33.9x + 130, 0.43	0.0032	93.8 ± 13.8	0.46
Cannabigerol (CBG)	0.657 ± 0.275	0.57	1.27 ± 0.217	0.85
CBG acid (CBGA)	y = −3.31x + 8.6, 0.58	0.0003	6.04 ± 1.07	0.82
Δ^9^-THC*eq*:CBD*eq*^x^	0.678 ± 0.0387	0.18	y = 0.0980x + 0.76, 0.26	0.030
Cannabinol (CBN)	UDL^w^	−	UDL	−
Alpha pinene	UDL	−	0.214 ± 0.0593	0.91
Beta pinene	0.235 ± 0.0672	0.37	0.440 ± 0.124	0.067
Myrcene	y = −4.16x + 6.2, 0.38	0.0089	y = −2.47x + 3.5, 0.36	0.0082
Limonene	y = −0.788x + 1.3, 0.34	0.014	1.57 ± 0.423,	0.058
Linalool	0.274 ± 0.0703	0.27	y = −0.147x + 0.22, 0.53	0.0010
Terpineol	0.254 ± 0.0655	0.32	0.379 ± 0.108	0.26
Caryophyllene	2.42 ± 0.746	0.28	y = 0.520x + 1.1, 0.35	0.0092
Humulene	0.892 ± 0.324	0.42	0.403 ± 0.0842	0.39
Fenchol	y = −0.118x + 0.22, 0.32	0.018	0.257 ± 0.0653	0.52
Guaiol	0.801 ± 0.100	0.17	y = 0.251x + 0.46, 0.31	0.016
Alpha-bisabolol	0.677 ± 0.181	0.26	0.355 ± 0.104	0.14
Total terpenes	y = −7.25x + 14, 0.38	0.0081	y = −2.72x + 9.2, 0.22	0.049

*^z^Parameters with UV treatment effects (P ≤ 0.05) are presented as equations and R^2^.*

*^y^Means ± SD are presented for parameters without UV treatment effects.*

*^x^The total equivalent cannabinoids are annotated with: eq, where Δ^9^-THCeq = (Δ^9^-THCA × 0.877) + Δ^9^-THC, CBDeq = (CBDA × 0.877) + CBD.*

*^w^Under detection limit of 0.5 mg⋅g^–1^ of inflorescence DW.*

### Responses of Inflorescence Yield, Apparent Quality, Cannabinoid and Terpene Concentrations to UV

**FIGURE 10:**
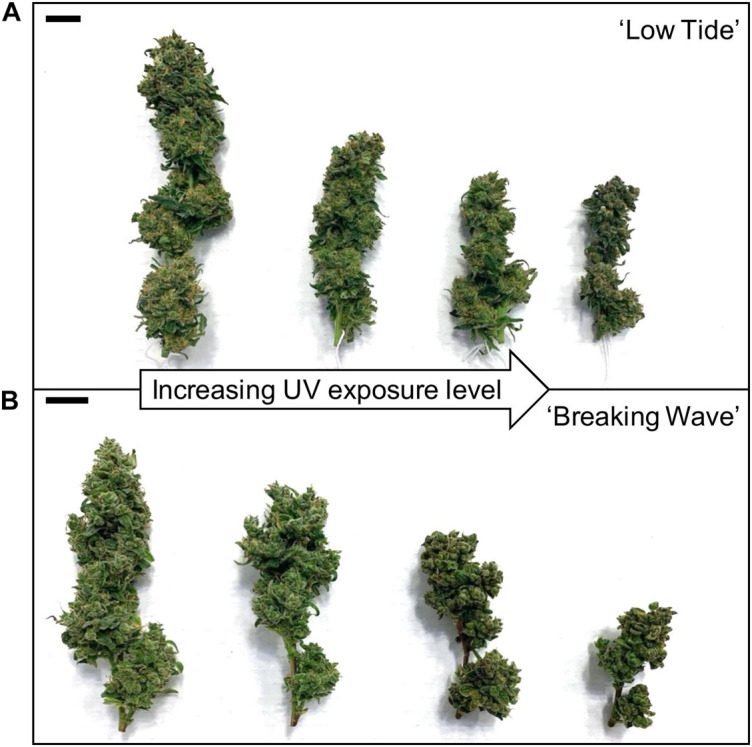
The apical inflorescences of representative **(A)** ‘Low Tide’ and **(B)** ‘Breaking Wave’ *Cannabis sativa* plants grown under (from left to right) minimum, low, moderate, and high UV exposure levels. Images were taken at harvest (i.e., 9 weeks after the initiation of UV treatments). The black scale bar at the upper left of each image is 2.0 cm.

**FIGURE 11:**
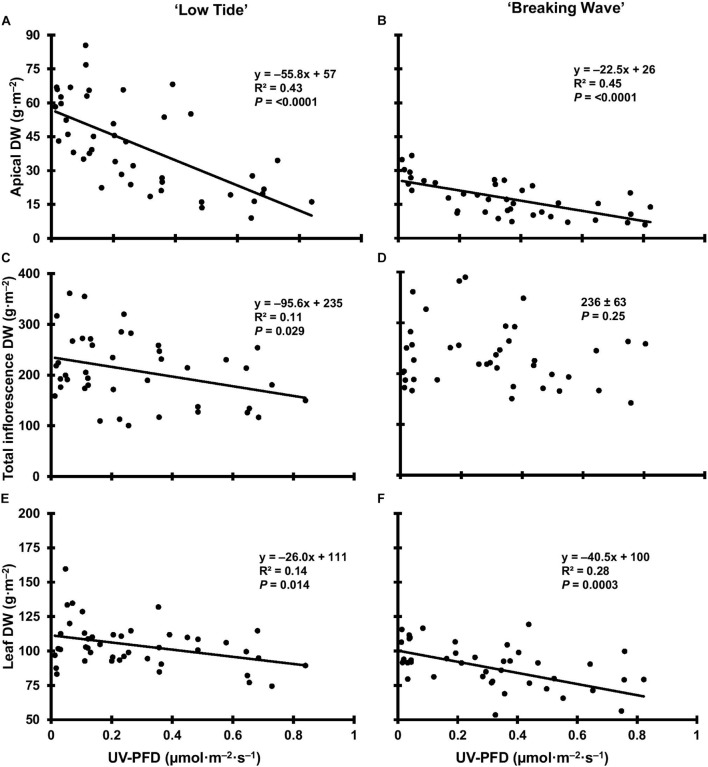
The apical inflorescence dry weight (DW) of *Cannabis sativa* ‘Low Tide’ **(A)** and ‘Breaking Wave’ **(B)**, the total inflorescence DW of ‘Low Tide’ **(C)** and ‘Breaking Wave’ **(D)** and the leaf DW of ‘Low Tide’ **(E)** and ‘Breaking Wave **(F)** in response to increasing UV-PFD. Each datum is a single plant.

The most discernable UV exposure effects on inflorescences were differences in the size of the apical inflorescences (Figure 10). The apical inflorescence DW decreased linearly by 78% and 69% in LT and BW, respectively, from the lowest to highest UV-PFD (Figures 11A,B). However, the reduction in apical inflorescence DW under increasing UV exposure only translated to reductions in total inflorescence DW in LT (Figures 11C,D). Approximately 60% of the reduction of the total inflorescence DW in LT at the highest vs. lowest UV exposure levels (a 32% reduction) arose from decreases in the DW of the apical inflorescences. The leaf DW were 19% and 32% lower under highest vs. lowest UV-PFD in LT and BW, respectively (Figures 11E,F); and there were no UV treatment effects on stem DW in either cultivar (Table 3).

**FIGURE 12:**
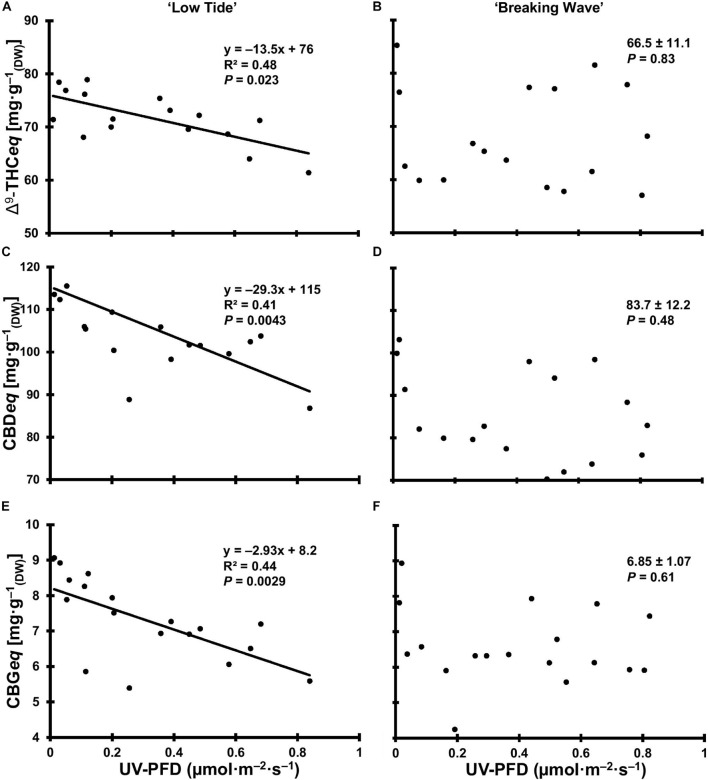
The total equivalent cannabinoid concentrations of Δ^9^-tetrahydrocannabinol (Δ^9^-THC*eq*) of *Cannabis sativa* ‘Low Tide’ **(A)** and ‘Breaking Wave’ **(B)**, cannabidiol (CBD*eq*) of ‘Low Tide’ **(C)** and ‘Breaking Wave’ **(D)** and cannabigerol (CBG*eq*) of ‘Low Tide’ **(E)** and ‘Breaking Wave’ **(F)** in response to increasing UV-PFD. Each datum is a single plant.

At the minimum UV exposure levels, the concentrations of the acid and neutral forms of both Δ^9^-THC and CBD and the ratio of Δ^9^-THC to CBD were within the normal range for each of these cultivars grown in the same production environment (without UV) according to the cultivator (personal communication). The effects of UV exposure on the apical inflorescence secondary metabolite composition varied between the two cultivars (Table 3 and Figure 12). Graphical representations of the best fit models for minor cannabinoids and terpenes that had UV-exposure treatment effects in at least one cultivar are also presented Supplementary Figures 1–12. In LT, the concentrations of Δ^9^-THCA, CBDA and CBGA decreased linearly by 15%, 21%, and 31%, respectively, as UV-PFD increased from lowest to highest; with concomitant reductions in Δ^9^-THC*eq*, CBD*eq*, and CBG*eq*. As UV-PFD increased from lowest to highest, the concentrations of myrcene, limonene, fenchol all decreased in LT, resulting in a combined 41% decrease in the total terpene content. In BW, the Δ^9^-THC concentration was 1.6 times higher and the ratio of Δ^9^-THC*eq* to CBD*eq* was 10% higher under the highest vs. lowest UV-PFD. The myrcene and linalool concentrations decreased while caryophyllene and guaiol concentrations increased with increasing UV-PFD, resulting in a combined 24% decrease in the total terpene content in BW at the highest vs. lowest UV-PFD.

## Discussion

Both LT and BW cultivars would be categorized as chemotype II because they have relatively balanced ratio of Δ^9^-THC to CBD (Small and Beckstead, 1973). However, they demonstrated disparate morphological attributes: LT had a relatively compact phenotype with wide leaflets and BW had a relatively spindly phenotype with narrow leaflets. Each cultivar responded to UV exposure with different magnitudes of severity but, in the majority of the parameters that had UV treatment effects, increasing UV exposure resulted in distress-type responses [i.e., damage to plant growth and health following a strong stress event (Hideg et al., 2013)] that would be generally unfavorable for commercial cannabis production.

### UV Radiation Alters Cannabis Morphology and Physiology

Leaf morphology demonstrated substantial plasticity in response to UV radiation exposure throughout the 9-week flowering stage. The first observed morphological response to UV was upward curling of leaflet margins during the first week of UV exposure. Upward leaf curling was most evident under higher UV-PFDs, and it occurred primarily on the youngest leaves (i.e., that developed in the vegetative stage, just prior to UV exposure). Upward leaf curling under UV stress is not a commonly-reported morphological response, although it has been observed in cotyledons of canola (Wilson and Greenberg, 1993). Upward leaf curling has been a more commonly- reported response to pathogen infection in some crops (Taliansky et al., 2003; Halldorson and Keller, 2018) and to various physiological stresses in tomato, including light stress (Powell et al., 2014). The recently-developed leaves in the present study may also have lacked the acclimative plasticity of the leaves that later developed under UV exposure, which exhibited more typical UV-induced morphology responses such as epinasty, reduced leaf size and increased leaf thickness indicated by SLW (Wilson, 1998; Searles et al., 2001; Zlatev et al., 2012; Fierro et al., 2015). Further, the apparent increase in leaf shine shortly after the initiation of UV exposures indicates an accumulation of epicuticular wax, which is a common response to UV exposure in other crops (Steinmüller and Tevini, 1985; Cen and Bornman, 1993; Fukuda et al., 2008; Valenta et al., 2020). All of these observed morphological responses to UV exposure may reduce the potential for damage to the photosynthetic machinery.

UV radiation accelerated plant senescence (i.e., deterioration with age) symptoms in both inflorescence and foliar tissues. Female inflorescence maturation can normally be characterized by carpel swelling and the transition of stigmas from white to brown in the final days before harvest (Punja and Holmes, 2020). Although the number of days between the initiation of the 12-h photoperiod and appearance of inflorescences was unaffected by UV exposure (data not shown), plants exposed to higher UV-PFDs exhibited earlier stigma browning (Figure 4). It is unknown if premature stigma senescence has any knock-on effects on other inflorescence development parameters, such as production of secondary metabolites. However, since the rate of stigma browning depended on UV exposure levels, this attribute could not be used reliably to determine the “optimum harvest maturity” for the plants in this trial.

Foliar chlorophyll content is often negatively correlated to UV exposure level (Neugart and Schreiner, 2018). Both cultivars showed increasing SLW with increasing UV exposure; similar to cannabis exposed to high PAR intensity (Rodriguez-Morrison et al., 2021a). While the CCI levels measured in this study were within the ranges of cannabis leaves reported by others (Caplan et al., 2017; Yep et al., 2020; Rodriguez-Morrison et al., 2021a), there were only UV treatment effects on CCI in the upper canopy leaves of BW. However, LT showed relatively greater increases in SLW with increasing UV exposure. Since foliar thickness affects a leaf’s optical properties, hence CCI measurements (Parry et al., 2014), LT’s enhanced increases in leaf thickness may have offset any UV-induced reductions in chlorophyll concentration on a biomass basis (e.g., mg⋅g^–1^) in this cultivar. While lower vs. upper canopy leaves in indoor-grown cannabis may have lower CCI, regardless of plant age (Rodriguez-Morrison et al., 2021a), the reductions in lower-canopy chlorophyll content with increasing UV-PFD in both cultivars may be another indication of the UV damage done to young leaves at onset of UV exposure. This damage eventually manifested as higher rates of early leaf-drop and increased leaf chlorosis at harvest in plants exposed to higher UV-PFDs. Both of these phenomena have also been observed in UV-stressed sweet basil (Dou et al., 2019). Nitrogen from lower canopy leaves is normally remobilized to more active upper canopy foliage as plants age (Havé et al., 2017); this appeared to be accelerated by UV exposure given the reductions in lower-canopy CCI after only 3 weeks of exposure. Foliar necrosis is also a commonly-observed symptom of UV damage in many species (Maffei and Scannerini, 2000; Zhao et al., 2003; Dou et al., 2019). While the severity of most of the observed UV stress responses increased with increasing UV exposure, necrotic patches were observed on upper canopy leaves that were predominantly exposed to intermediate UV-PFDs (primarily in LT) in the latter weeks of the trial. The acclimation (e.g., epinasty, curling, reduced size) of leaves exposed to the highest UV-PFDs may have mitigated foliar damage, while the leaves grown under intermediate UV-PFDs may not have been sufficiently acclimated for long-term UV exposure. Unstressed leaves normally have F_v_/F_m_ values of ≈0.8 (Björkman and Demmig, 1987). While the reductions in F_v_/F_m_ in upper canopy leaves of both cultivars were similar to cannabis plants exposed high PAR intensity in Rodriguez-Morrison et al. (2021a), the opposite effects of increasing UV vs. PAR radiation on NCER is strongly indicative of UV-induced damage to the photosynthetic machinery.

Lydon et al. (1987) reported no UV treatment effects on the cannabis morphology and physiology parameters they measured, which is in stark contrast to the copious morphological and physiological UV-induced stress responses observed in the present study. Evidently, the plants in the present study were subjected to more efficacious UV radiation treatments than in Lydon et al. (1987) despite similar reported maximum biologically-effective UV doses in both studies. This may be partly due to the shorter-wavelength UV spectrum in the present study. Further, the plants in Lydon et al. (1987) may have experienced lower than reported doses due to rapid UV-induced reductions of UVB transmissivity of the cellulose acetate filters they used to eliminate UVA and PAR wavelengths from their UV spectrum treatments (Middleton and Teramura, 1993). Additionally, their plants grew for several months under greenhouse conditions (likely including some UV) prior to exposure to UV treatments, whereas there was no UV exposure of the plants prior to initiation of the UV treatments in the present study. Therefore, light history (e.g., spectrum and intensity) and plant age may affect how plants to acclimate to new UV stresses.

### UV Radiation Suppresses Cannabis Growth and Yield

While increasing UV radiation exposure suppressed overall vegetative plant growth (e.g., height and growth index) in both cultivars, the responses were more severe in LT than BW. However, these are in contrast with the UV-induced reductions in foliar biomass, which were substantially greater in BW. This was particularly surprising given that there were no consequent reductions in total inflorescence biomass in BW. In fact, despite some leaf senescence observed in both cultivars, harvest index – which is the ratio of inflorescence DW to total aboveground DW – went up by ≈10% in BW and went down by ≈10% in LT as UV-PFD increased from lowest to highest. Under low UV exposure, the harvest index for both cultivars was ≈0.6, which was similar to a different cultivar grown under the same PPFD in the same production system without UV (Rodriguez-Morrison et al., 2021a). Given that there were no UV exposure effects on inflorescence DW in BW, earlier and/or elevated foliar senescence in BW may have contributed to its relatively elevated harvest index.

Reduced aboveground biomass and lower yields are commonly observed effects of UV radiation on some other plant species (Teramura et al., 1990; Fiscus and Booker, 1995; Caldwell et al., 2003; Liu et al., 2005). The UV-induced alterations in leaf morphology and physiology probably contributed to the general reductions in growth and overall biomass in both cultivars. For example, reduced leaf area is a typical response to radiative stresses such as high PAR intensity and UV exposure (Wargent and Jordan, 2013; Poorter et al., 2019). In the present study, the reductions in individual leaf size, total foliar biomass, and leaf-level NCER with increasing UV exposure, would have limited the plants’ capacity to convert PAR into biomass (Kakani et al., 2003; Zlatev et al., 2012).

Total inflorescence DW and the proportion of that DW which is comprised of apical tissues are two major considerations for commercial cannabis production. The apical proportion may be of particular interest since these tissues are normally considered premium quality due to their relatively large size and potentially higher cannabinoid concentrations compared to higher-order (i.e., on lower branches) inflorescences (Namdar et al., 2018). Despite the UV-induced limitations to biomass accumulation seen in both cultivars, increasing UV exposure only reduced inflorescence DW in LT. Within this context, the various growth habits of common indoor-grown cannabis cultivars may influence their yield responses to UV stress. In the present study, BW and LT had disparate whole-plant reproductive macro-morphology (i.e., the distribution of inflorescence biomass within the canopy) under normal indoor conditions. For example, under minimum UV exposure, the apical inflorescence comprised 24% of the total inflorescence DW in LT compared to only 11% in BW. Apparently, growth habit may have predisposed BW’s mitigation of UV-induced yield reductions by partitioning relatively more inflorescence biomass to positions farther away (i.e., more protected from the UV) from the top of the plant. However, while this may be a self-protective response to reduce UV exposure to reproductively important (from an ecological sense) tissues, it still came at commercially-objectionable reductions in inflorescence quality, such as visually unappealing morphology (Figure 10).

To prevent UV-induced yield losses, such as are reported in the present study, it is conceivable that cannabis plants could be exposed to UV only after the majority of vegetative growth has completed [i.e., a few weeks after the visual appearance of inflorescences (Potter, 2014)]. This strategy would shorten the accumulated period of exposure to UV stress and may minimize some UV-induced foliar acclimations that could inhibit biomass accumulation. However, there is a risk that later-term UV exposure might also sufficiently stress unacclimated foliar tissues to provoke rapid-onset whole-plant senescence before the inflorescences reach optimum maturity. This strategy warrants further exploration.

### UV Radiation Alters the Secondary Metabolite Composition of Cannabis Inflorescences

The most economically relevant cannabinoids (i.e., Δ^9^-THC and CBD) are predominantly found in their acid forms in mature female inflorescence tissues, which are converted to the psychoactive and medicinal neutral forms through decarboxylation (Eichler et al., 2012; Zou and Kumar, 2018). The neutral forms also exist in relatively low quantities in the fresh inflorescences and tend to increase in proportion to the acid forms as the inflorescences mature (Aizpurua-Olaizola et al., 2016). While the Δ^9^-THC concentration increased in BW with increasing UV-PFD, it was a relatively small proportion of the Δ^9^-THC*eq*; maximized at 3.3% at the highest UV-PFD. Further, CBN was undetectable in the inflorescences, which is an indicator that the crops were not past peak maturity at the time of harvest since Δ^9^-THC naturally degrades to CBN (Russo, 2007). There were no UV-induced enhancements to Δ^9^-THC*eq*, CBD*eq*, and CBG*eq* in either cultivar. These results are consistent with a recent study that found no UV treatment effects on Δ^9^-THC*eq* content in a Δ^9^-THC-dominant cultivar (Llewellyn et al., 2021), but contrast with studies on older genotypes (Pate, 1983; Lydon et al., 1987). For example, Lydon et al. (1987) found that inflorescence Δ^9^-THC concentrations increased linearly from 32 to 25 mg⋅g^–1^ in greenhouse-grown cannabis as UV exposure increased from their no-UV control up to biologically-effective UV doses (based on Caldwell, 1971) of 13.4 kJ⋅m^–2^⋅d^–1^. These contrasting results may be due to the disparate growing conditions (both before and during UV exposure), plant age at the time of UV exposure, and the relative magnitude of cannabinoid concentrations. Further, while the proportional increases in Δ^9^-THC content (28%) presented in Lydon et al. (1987) appeared to be substantial, the magnitude of their increase (i.e., only 7 mg⋅g^–1^) is probably inconsequential in the context of cannabinoid composition in modern genotypes which can have Δ^9^-THC concentrations that exceed 200 mg⋅g^–1^ (Dujourdy and Besacier, 2017).

Pate (1983) reported an increase in the ratio of Δ^9^-THC to CBD in inflorescence tissues of cannabis ecotypes grown in global positions with naturally higher UV exposures, which suggests that the production of Δ^9^-THC may be upregulated and CBD downregulated as adaptations (i.e., over multiple generations) to the localized environment. However, the results of the present study do not support this trend, at least as an acclimation response to UV stress of a single generation. Additionally, De Meijer et al. (2003) showed that cannabinoid profiles are largely genetically predetermined (e.g., a CBD-dominant cultivar is lacking the genetic predisposition to generate abundant Δ^9^-THC). This favors the concept that the upregulation of Δ^9^-THC under UV stress may be an adaptive response (i.e., over generations) rather than an acclimation response (i.e., during a single production cycle). Over the past few decades, there have been radical increases in inflorescence cannabinoid concentrations, which is often attributed to intensive breeding programs (Chouvy, 2015; Dujourdy and Besacier, 2017; Aliferis and Bernard-Perron, 2020) and the “sinsemilla” cultivation method that eliminates seeds and chiefly produces high potency female inflorescences (ElSohly et al., 2016). Thus, these factors may have a larger impact on cannabis inflorescence cannabinoid composition in indoor production than environmental factors such as UV stress.

While cannabinoids comprise the primary psychoactive and medicinal compounds in cannabis inflorescences, volatile terpenes are also economically valuable; both for the aromas that influence consumer preference and potential medicinal properties (Nuutinen, 2018; Booth and Bohlmann, 2019). UV exposure equivocally altered the terpene composition in the present study, with disparate responses within the different terpenes and between cultivars. However, total terpene concentrations in both cultivars decreased linearly with increasing UV exposure, which would tend to depreciate the overall quality of aromas and extracts (McPartland and Russo, 2001; Nuutinen, 2018).

While UV exposure did not result in any economically relevant increases in cannabinoid or terpene concentrations in cannabis inflorescences under the conditions of the present study, UV radiation has been shown to increase concentrations of UV-absorbing secondary metabolites (e.g., flavonoids and phenolic compounds) in many species (Huché-Thélier et al., 2016; Robson et al., 2019), including economically important essential oil producing crops (Schreiner et al., 2012; Neugart and Schreiner, 2018). However, UV-induced increases in secondary metabolite concentrations are often concurrent with biomass reductions (Fiscus and Booker, 1995; Caldwell et al., 2003). This paradox must be evaluated when considering the use of UV radiation to manipulate secondary metabolite composition in indoor cannabis production, since the simultaneous yield reduction may offset any improvements in secondary metabolite composition.

Compared to the UV spectra employed in most other studies, the biologically effective doses in the present study were dramatically higher for a given photon flux density due to the very short peak wavelength of the UV LEDs. In fact, ≈70% of the UV photon flux were at wavelengths below 290 nm, and thus outside of the solar spectrum that plants would naturally be exposed and adapted to Nikiforos et al. (2011). Therefore, cannabis may respond dramatically differently to UV from slightly longer wavelength LEDs (e.g., 300 to 315 nm).

### Implications for UV Use in Indoor Cannabis Production and Future Research Directions

This study provided insight into the sensitivity of cannabis to relatively short-wavelength UVB radiation (including a small proportion of UVC) and long-term UV exposure. Increasing UV exposure levels generally had negative impacts on cannabis plant growth, yield, quality, and secondary metabolite composition. The plants exhibited primarily distress-type responses to UV radiation, even at low exposure levels; no amount of UV exposure resulted in substantial increases of cannabinoid concentrations. While none of the UV exposure levels in the present study would have been commercially beneficial, results from studies in other species (Huché-Thélier et al., 2016; Neugart and Schreiner, 2018; Höll et al., 2019; Robson et al., 2019) indicate a strong potential for there being UV treatment protocols – as yet unidentified through rigorous scientific investigation and reporting – that could enhance secondary metabolite concentrations in cannabis. Further research is required to determine if there is a combination of UV spectrum, intensity and time of application that would have commercially beneficial effects in cannabis production. The range of tested cannabis cultivars should also be expanded to cover a broader range of chemotypes and growth habits.

When making the decision to utilize UV wavelengths (as with any production technology) in indoor cannabis production, the positive crop outcomes must outweigh factors related to the cost of deploying the technology including infrastructure and energy costs, fixture lifespan, and health risks that UV radiation could pose to employes. While UVB LEDs in particular (Kusuma et al., 2020) and UV lighting technologies in general are much less energy efficient than modern horticultural PAR fixtures (Nelson and Bugbee, 2014; Radetsky, 2018), UV fluence rates are also typically many times lower than the PAR spectrum. The functional lifespans of UVB LEDs are currently much lower (Kebbi et al., 2020) than common horticultural LEDs (Kusuma et al., 2020); potentially leading to relatively rapid degradation in fluence rates over time. Given that plant responses in the present study were closely tied to the UV exposure level, fixture degradation could lead to inconsistencies between sequential crops, which is an important parameter in the indoor cannabis production industry.

Overall, it is still possible that the alternate UV treatment protocols may have more positive results in the controlled environment production of modern, drug-type cannabis cultivars; for example: longer wavelength and less energetic spectra (Hikosara et al., 2010) and shorter-term (e.g., proximal to harvest maturity) exposure (Johnson et al., 1999; Martínez-Lüscher et al., 2013; Huarancca Reyes et al., 2018; Dou et al., 2019). Future research could seek to promote eustress responses in cannabis secondary metabolite concentrations while minimizing distress responses (e.g., yield reductions) by using less energetic UV spectra and/or different daily exposure protocols than were used in the present study. The effects of cannabis plants grown under different lighting histories should also be investigated to determine the ideal developmental stage for UV exposure to achieve the desired effects in both yield and quality.

## Conclusion

Long-term exposure of various intensities of relatively short-wavelength UV radiation had generally negative impacts on cannabis growth, yield, and inflorescence quality. By studying two cultivars with similar cannabinoid profiles, we found some differences in phenotypic plasticity in the temporal dynamics in morphology, physiology, yield, and quality responses to UV exposure level. For the first time this paper described the visible symptoms caused by UVB stress on indoor cannabis plants. Importantly, as it was applied in this study, UV radiation provoked substantially reduced yield in one cultivar, reduced inflorescence quality in both cultivars, and had no commercially relevant benefits to inflorescence secondary metabolite composition. Therefore, potential for UV radiation to enhance cannabinoid concentrations must still be confirmed before UV can be used as a tool in cannabis production.

## Data Availability Statement

The raw data supporting the conclusions of this article will be made available by the authors, without undue reservation.

## Author Contributions

VR-M and DL performed the experiment and collected and analyzed the data. VR-M, DL, and YZ wrote and revised the manuscript. All authors contributed to the experimental design and approved the final manuscript.

## Conflict of Interest

The authors declare that the research was conducted in the absence of any commercial or financial relationships that could be construed as a potential conflict of interest.

## Publisher’s Note

All claims expressed in this article are solely those of the authors and do not necessarily represent those of their affiliated organizations, or those of the publisher, the editors and the reviewers. Any product that may be evaluated in this article, or claim that may be made by its manufacturer, is not guaranteed or endorsed by the publisher.

## Abbreviations

NCER: net carbon dioxide exchange rate
PPFD: photosynthetic photon flux
PFD: photon flux density
CCI: chlorophyll content index
SLW: specific leaf weight
LED: light-emitting diode
DLI: daily light integral
PAR: photosynthetically active radiation
DW: dry weight
SD: standard deviation
Δ^9^-THC: Δ^9^-tetrahydrocannabinol
Δ^9^-THCA: Δ^9^-tetrahydrocannabinolic acid
CBD: cannabidiol
CBDA: cannabidiolic acid
CBG: cannabigerol
CBGA: cannabigerolic acid
CBN: cannabinol
UV: ultraviolet
UVA: ultraviolet-A
UVB: ultraviolet-B
UVC: ultraviolet-C
Fv/Fm: variable to maximum chlorophyll fluorescence
TLI: total light integral
LT: ‘Low Tide’
BW: ‘Breaking Wave’
CB: culture basin
NIE: no increase in extent
NI: not investigated
UDL: under detection limit
UV-PFD: photon flux density of ultra-violet radiation.
