# Supplementary material for: Cannabis Inflorescence Yield and Cannabinoid Concentration Are Not Increased With Exposure to Short-Wavelength Ultraviolet-B Radiation
Source: Front Plant Sci. 2021 Nov 2;12:725078. doi: 10.3389/fpls.2021.725078 (PMC8593374; doi:10.3389/fpls.2021.725078)
Supplement: Supplementary file 1 [file Data_Sheet_1.PDF]

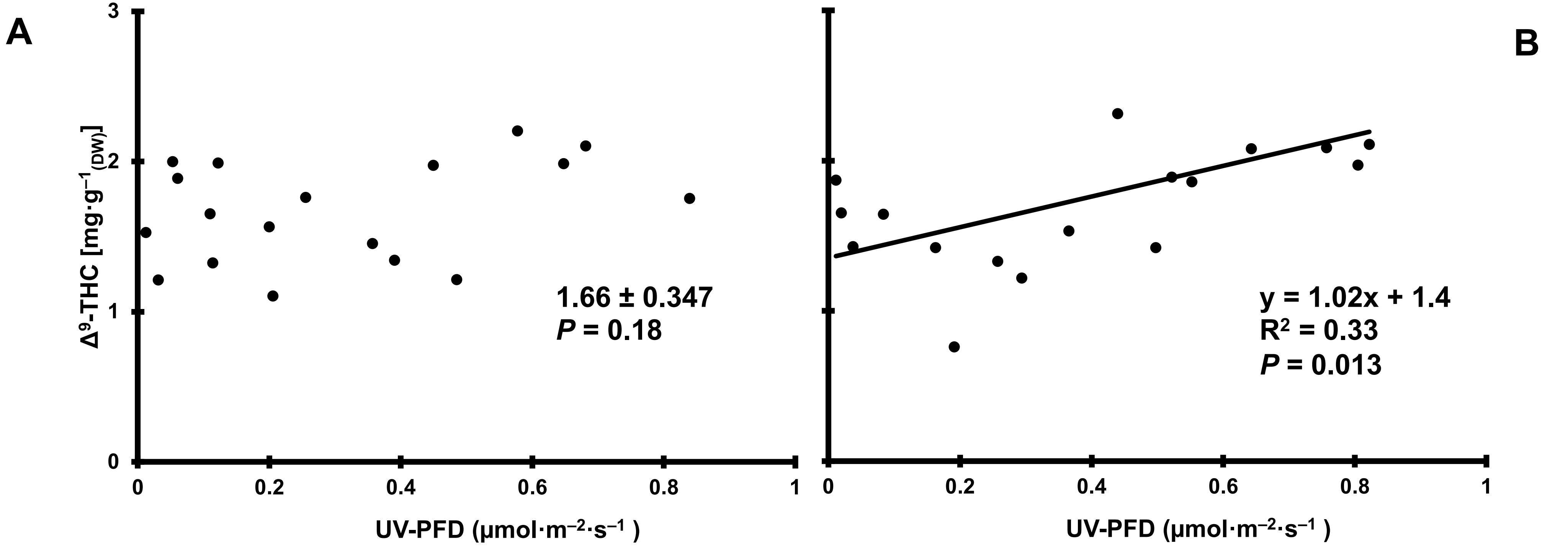

Figure S1. The response of Δ^{9}-tetrahydrocannabinol (Δ^{9}-THC) in 'Low Tide' **(A)** and 'Breaking Wave' **(B)** to UV-PFD.

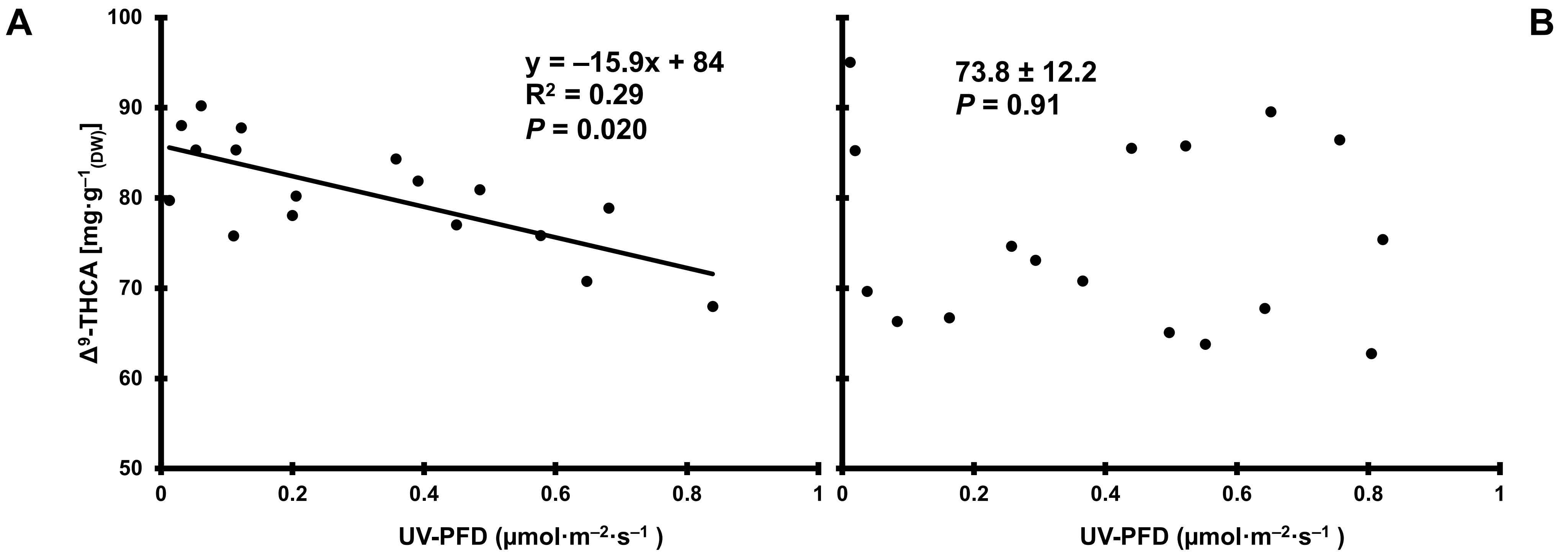

Figure S2. The response of Δ⁹-tetrahydrocannabinolic acid (Δ⁹-THCA) in ‘Low Tide’ **(A)** and ‘Breaking Wave’ **(B)** to UV-PFD.

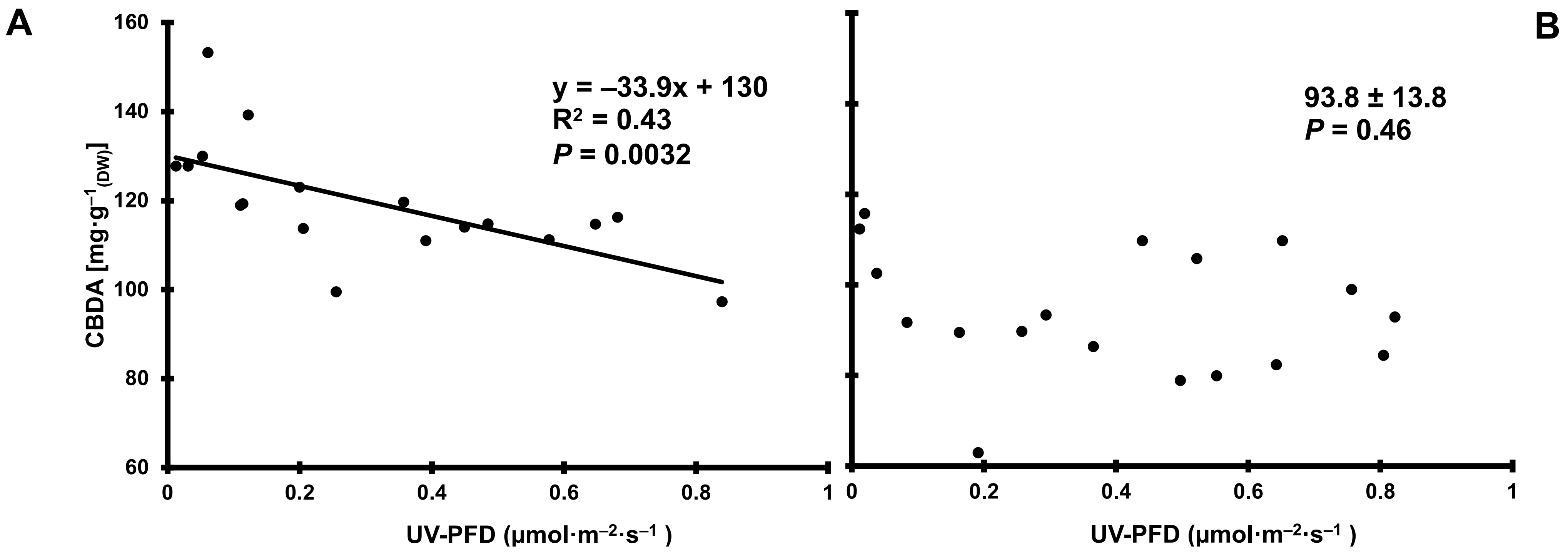

Figure S3. The response of cannabidiolic acid (CBDA) in 'Low Tide' **(A)** and 'Breaking Wave' **(B)** to UV-PFD.

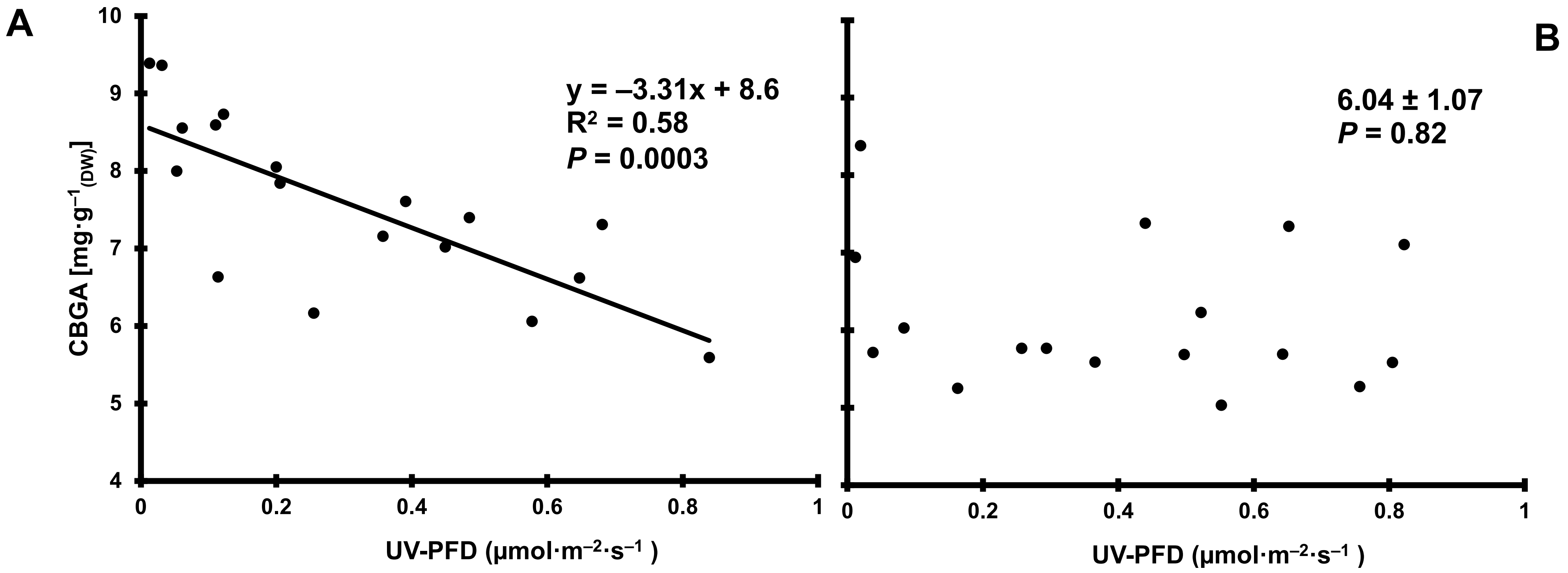

Figure S4. The response of cannabigerolic acid (CBGA) in 'Low Tide' **(A)** and 'Breaking Wave' **(B)** to UV-PFD.

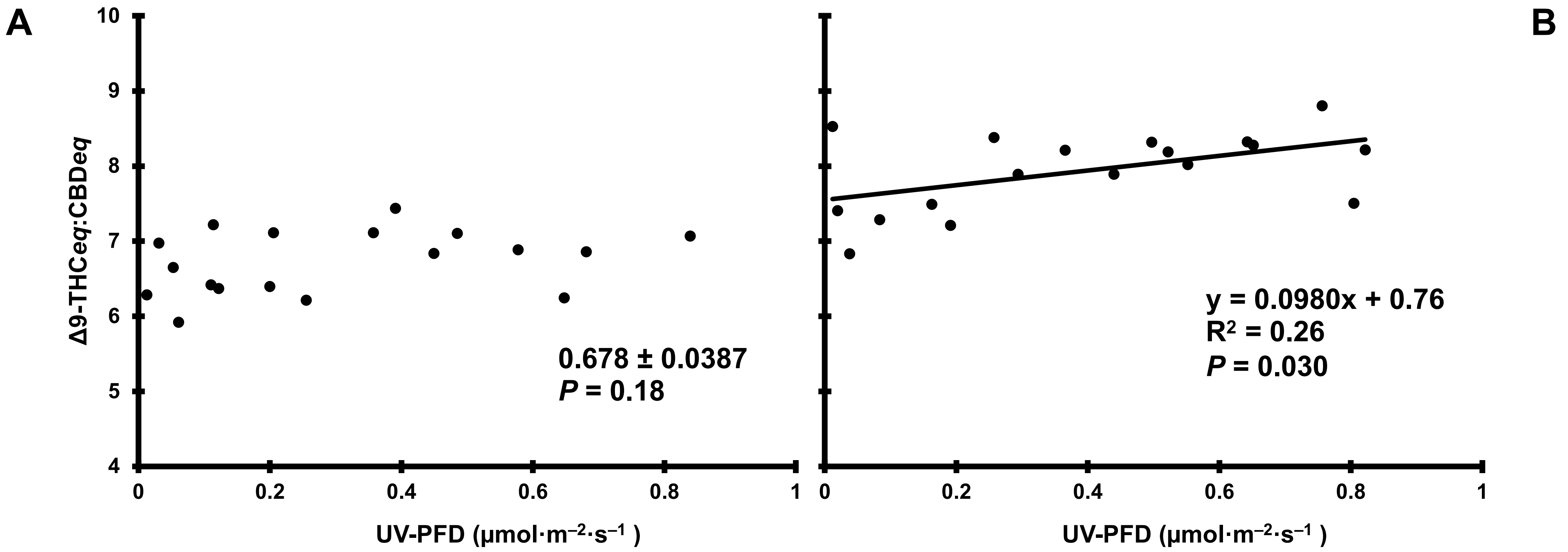

Figure S5. The response of the ratio of total equivalent Δ^9-tetrahydrocannabinol to total equivalent cannabidiol (Δ^9-THC*eq*:CBD*eq*) in 'Low Tide' **(A)** and 'Breaking Wave' **(B)** to UV-PFD.

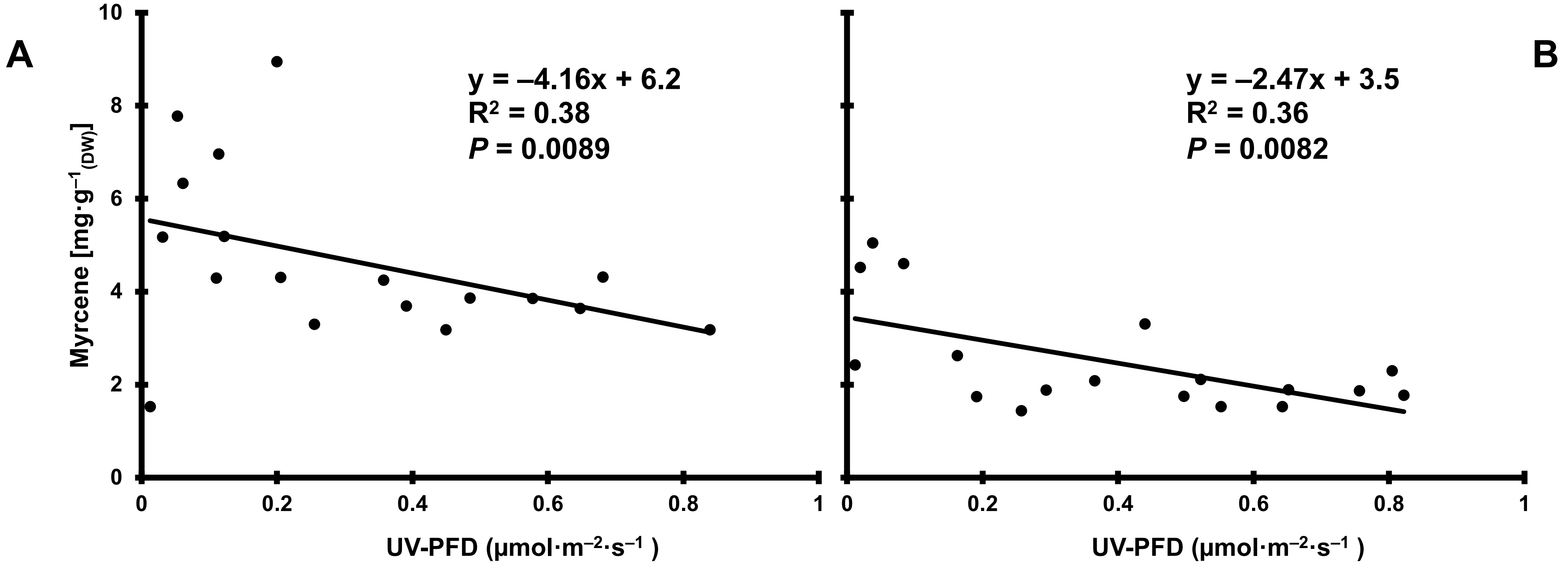

Figure S6. The response of myrcene in 'Low Tide' **(A)** and 'Breaking Wave' **(B)** to UV-PFD.

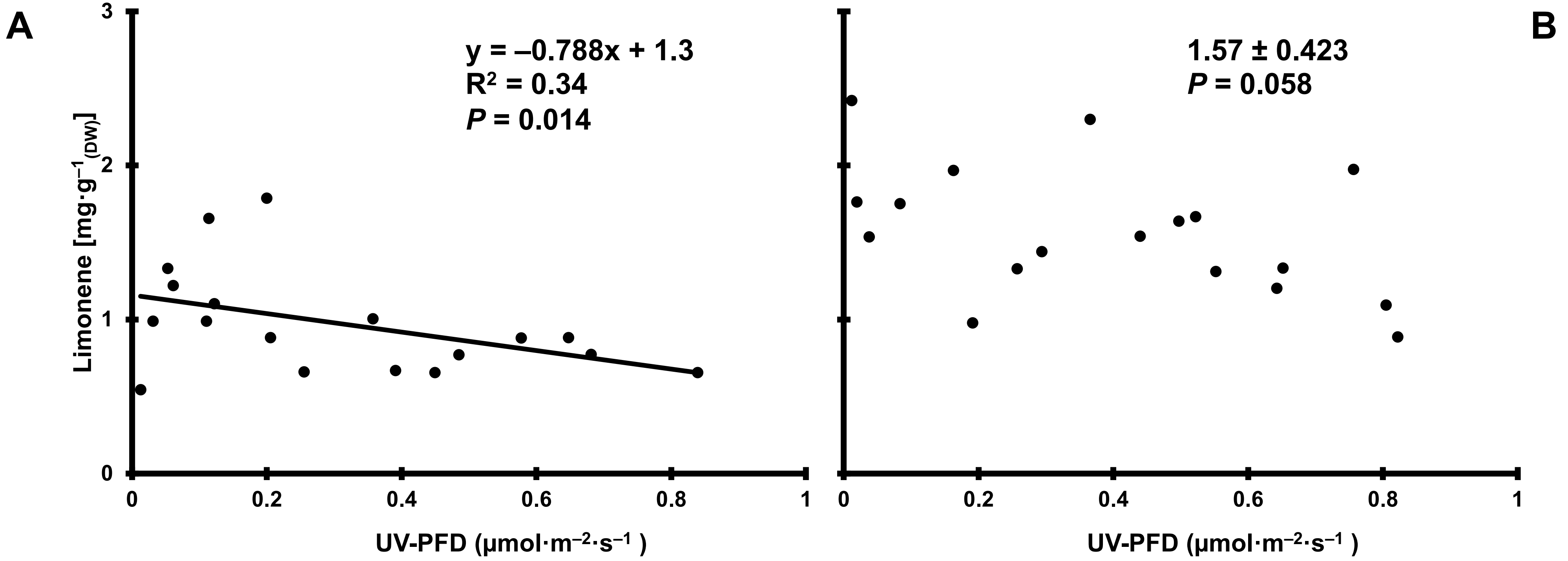

Figure S7. The response of limonene in 'Low Tide' **(A)** and 'Breaking Wave' **(B)** to UV-PFD.

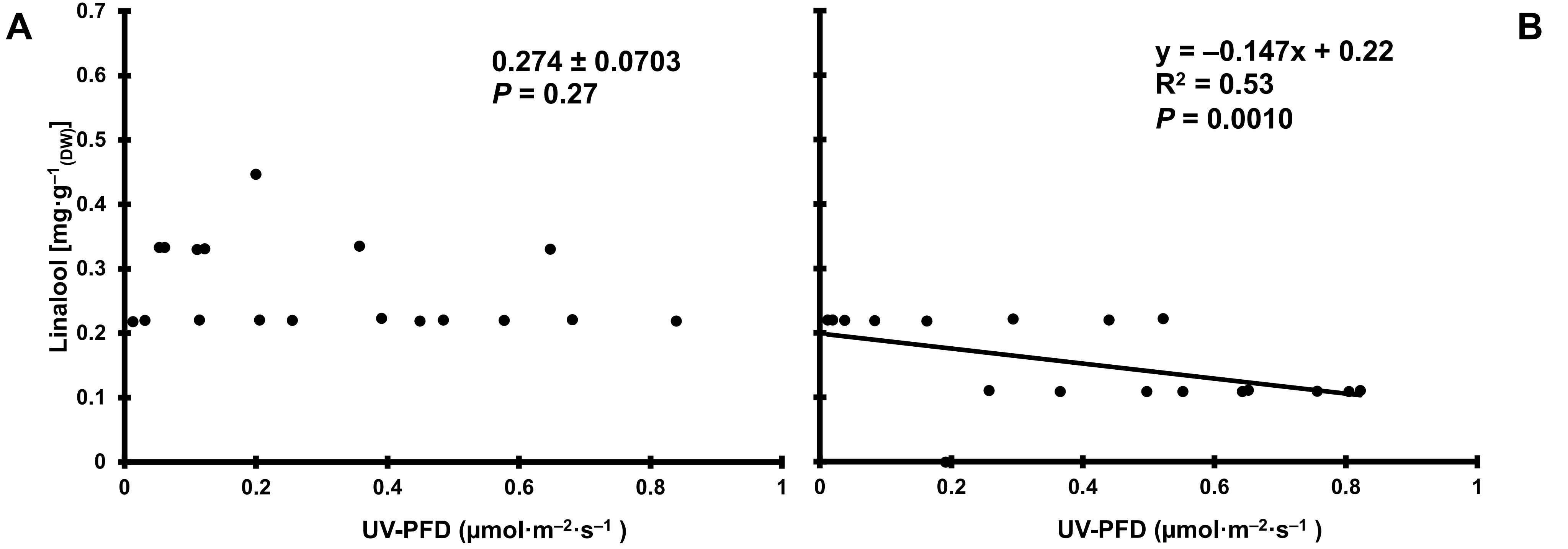

Figure S8. The response of linalool in 'Low Tide' **(A)** and 'Breaking Wave' **(B)** to UV-PFD.

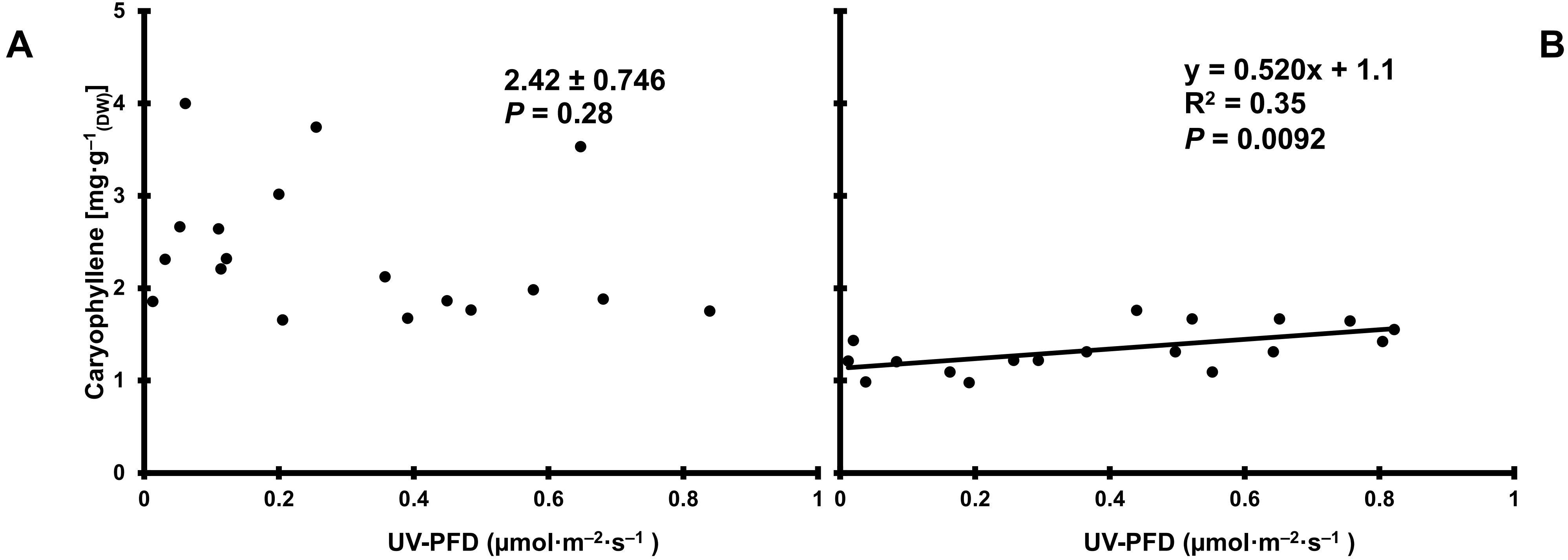

Figure S9. The response of caryophyllene in ‘Low Tide’ **(A)** and ‘Breaking Wave’ **(B)** to UV-PFD.

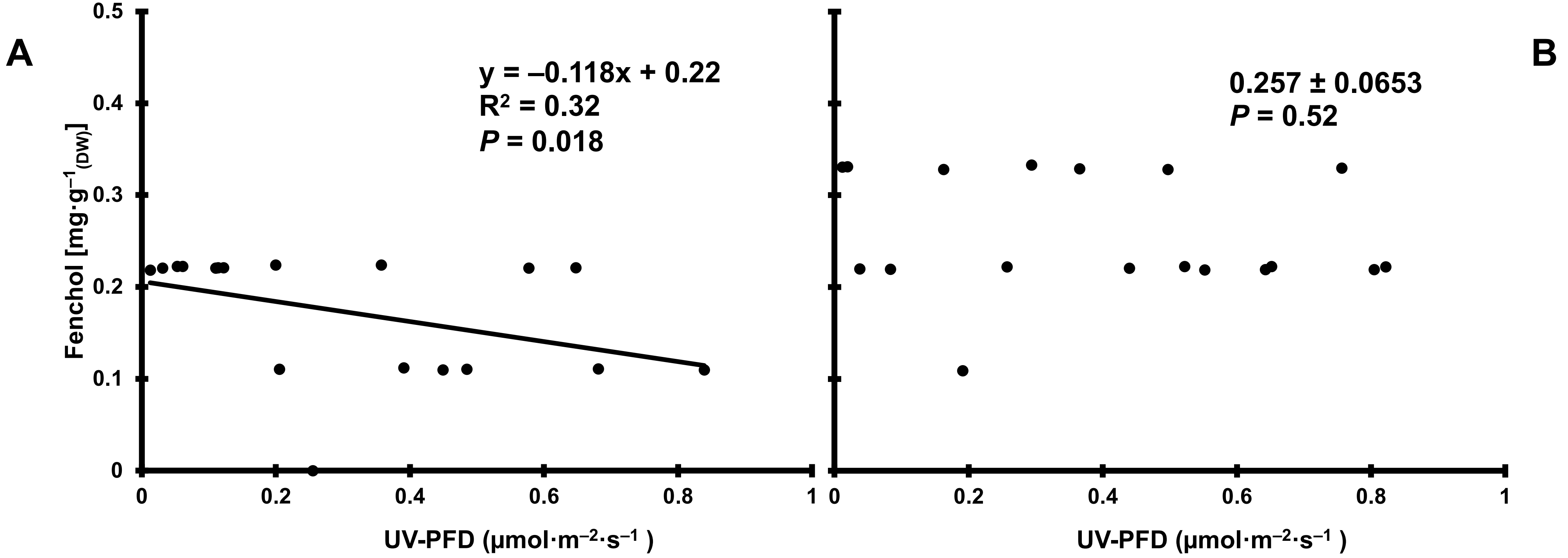

Figure S10. The response of fenchol in ‘Low Tide’ **(A)** and ‘Breaking Wave’ **(B)** to UV-PFD.

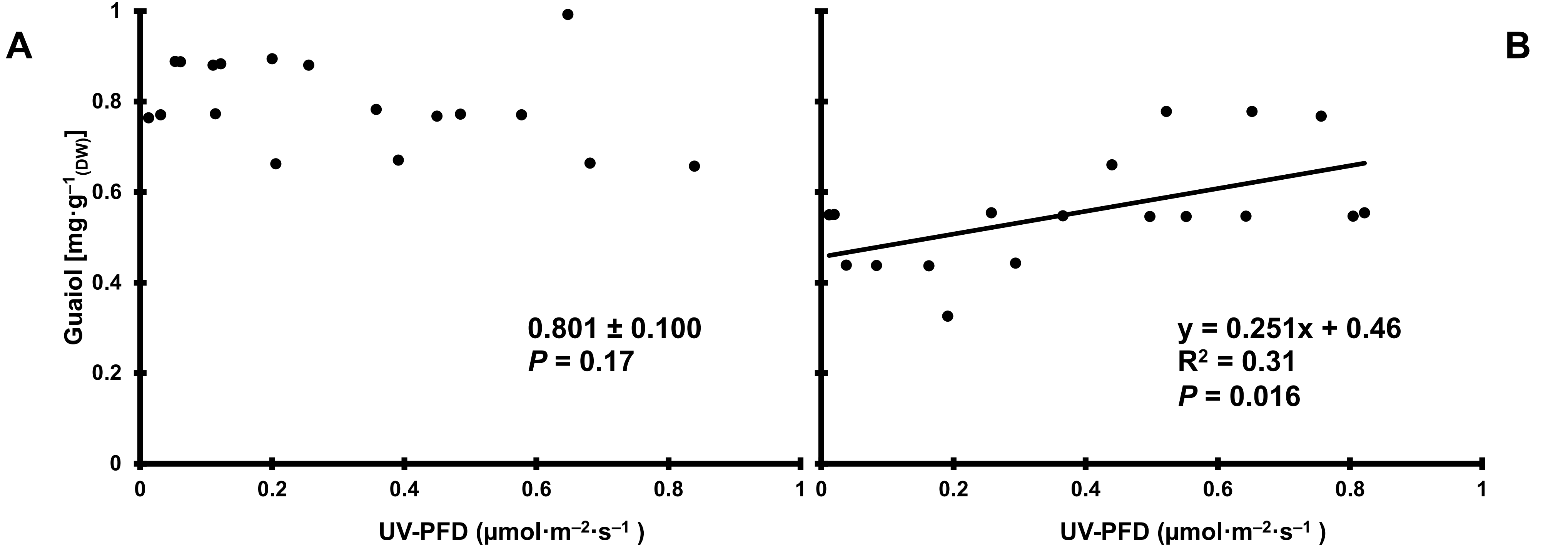

Figure S11. The response of guaiol in 'Low Tide' **(A)** and 'Breaking Wave' **(B)** to UV-PFD.

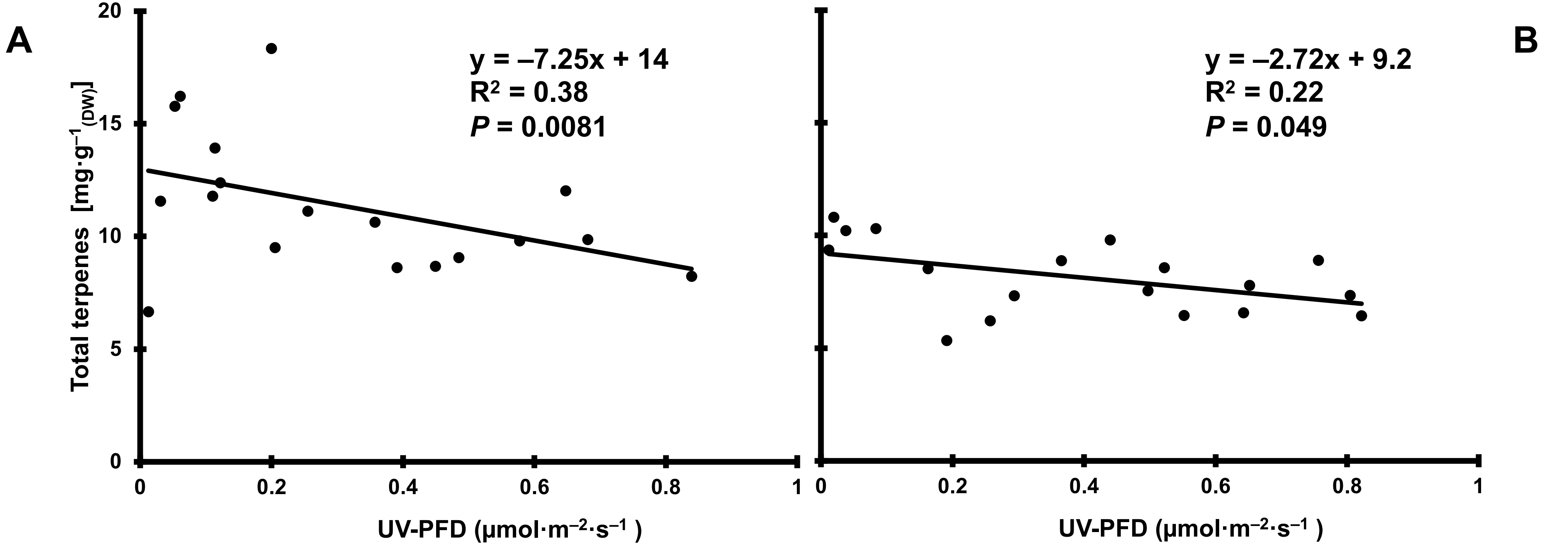

Figure S12. The response of total terpenes in 'Low Tide' **(A)** and 'Breaking Wave' **(B)** to UV-PFD.
